# Identification of new high affinity targets for Roquin based on structural conservation

**DOI:** 10.1093/nar/gky908

**Published:** 2018-10-08

**Authors:** Johannes Braun, Sandra Fischer, Zhenjiang Z Xu, Hongying Sun, Dalia H Ghoneim, Anna T Gimbel, Uwe Plessmann, Henning Urlaub, David H Mathews, Julia E Weigand

**Affiliations:** 1 Department of Biology, Technische Universität Darmstadt, Darmstadt 64287, Germany; 2 Department of Biochemistry and Biophysics and Center for RNA Biology, University of Rochester Medical Center, Rochester, NY 14642, USA; 3 Biophysical Mass Spectrometry Group, Max Planck Institute for Biophysical Chemistry, Göttingen 37077, Germany; 4 Bioanalytics, Institute for Clinical Chemistry, University Medical Center, 37073 Göttingen, Germany

## Abstract

Post-transcriptional gene regulation controls the amount of protein produced from a specific mRNA by altering both its decay and translation rates. Such regulation is primarily achieved by the interaction of *trans*-acting factors with *cis*-regulatory elements in the untranslated regions (UTRs) of mRNAs. These interactions are guided either by sequence- or structure-based recognition. Similar to sequence conservation, the evolutionary conservation of a UTR’s structure thus reflects its functional importance. We used such structural conservation to identify previously unknown *cis*-regulatory elements. Using the RNA folding program Dynalign, we scanned all UTRs of humans and mice for conserved structures. Characterizing a subset of putative conserved structures revealed a binding site of the RNA-binding protein Roquin. Detailed functional characterization *in vivo* enabled us to redefine the binding preferences of Roquin and identify new target genes. Many of these new targets are unrelated to the established role of Roquin in inflammation and immune responses and thus highlight additional, unstudied cellular functions of this important repressor. Moreover, the expression of several Roquin targets is highly cell-type-specific. In consequence, these targets are difficult to detect using methods dependent on mRNA abundance, yet easily detectable with our unbiased strategy.

## INTRODUCTION

The precise regulation of gene expression in response to internal or external signals is crucial for cell survival and the prevention of pathologies. In higher eukaryotes, post-transcriptional regulation, affecting both the mRNA and protein levels, leads to several layers of complexity, which are particularly necessary for the establishment of cell-type- and tissue-specific gene expression.

The central hubs for regulation at the RNA level are mRNAs. Early regulation steps influence the processing of pre-mRNAs into mature mRNA, with the regulation of splicing being of particular importance. After export to the cytoplasm, the localization, translation efficiency and stability of an mRNA are tightly controlled. Such regulation is primarily based on *cis*-acting elements in the untranslated regions (UTRs) of mRNAs. These elements are recognized by *trans*-acting factors, mainly RNA-binding proteins (RBPs) and non-coding RNAs, like microRNAs (miRNAs). Recognition is either sequence-based—typically short, single-stranded motifs—or shape-based, i.e. depending on the three-dimensional structure of the UTR (1). Precise knowledge of the type and number of regulatory elements in a UTR is necessary to understand how specific expression patterns are achieved and to predict changes in response to stress signals. While a variety of methods for the prediction and high-throughput identification of linear sequence motifs are established (2–4), structural elements that contribute to gene regulation are more difficult to detect and therefore less studied.

Recognizing this shortfall, several methods based on high-throughput sequencing have recently been developed to determine RNA structures *in vivo. In vivo* probing of RNA structures is based on cell-permeable chemicals with different reactivity for single- and double-stranded regions (5–8). In these cases, however, it is unclear whether an undetected region, i.e. one that is not accessible to the chemical, is actually double-stranded or bound by a protein. Complementary methods directly detect double-stranded regions by proximity ligation after cross-linking. These can either detect unbound double stranded regions with psoralen-induced cross-linking or protein-bound regions by UV-induced cross-linking in combination with immunoprecipitation of the desired protein (9,10). Such methods are promising for a comprehensive elucidation of the RNA structurome in the future, but they have certain limitations. One disadvantage is that all methods are limited to the transcripts (abundantly) present at the time of the experiment. Thus, cell-type-specific or stress-induced transcripts are not detected.

RNA secondary structure prediction provides an alternative approach to detecting specific structures that is independent of the expression level (11,12). Thus it is possible to query all human UTRs simultaneously for potential structure formation. Furthermore, base pairing probabilities provide additional information; highly probable pairs are more likely to be correctly predicted than less probable pairs (13,14). Similar to sequence conservation, evolutionary conservation of structures is an indicator of functional importance (15). Comparative analyses of structure conservation in UTRs of related species thus improve the accuracy of *de novo* discovery of new structured elements that function in gene regulation.

Nucleotide mutation patterns that maintain structure formation, e.g. compensatory mutations in stem regions, are called covariations. They are indicative of functional structures and can thus be used for the discovery of structured RNA. This principal was first exploited by the program QRNA, which used such mutational patterns as the signal to find conserved structures (16). Bioinformatic screens for assessing covariation in UTRs were successfully used to identify functional structured elements in bacterial mRNAs (17). In addition to the lower sequence content, the decisive advantage in bacteria is the availability of a large number and variety of genomes. The comparatively few mammalian genomes, however, are less diverse due to a closer evolutionary relationship. Therefore, covariation will be less useful for the discovery of conserved RNA structures in mammals, than it was within bacteria.

An alternative means to discover conserved structures, independent from mutation patterns, is folding stability. This was used by RNAz to find conserved RNA structures (18,19). RNAz uses a fixed input alignment, but sequence alignment is difficult for sequences of low nucleotide identity. As alternatives, Dynalign and Foldalign were both extended to discover conserved structures (20,21). These programs take two unaligned sequences and simultaneously fold and align the sequences to identify the lowest free energy conserved structure (22,23).

While most of the RBPs characterized so far recognize single-stranded sequences, Roquin proteins are distinctive for their specific recognition of the shape of stem loop structures (24,25). Mammals encode two highly similar paralogs, Roquin-1 and Roquin-2 (*RC3H1* and *RC3H2*), which seem to fulfill largely redundant functions. They share >80% of sequence identity in their N-terminal region, coding a RING domain (really interesting new gene) and two RNA-binding domains: the ROQ domain, which is unique for Roquin proteins and a CCCH-type ZnF (zinc finger). The C-terminal region is predicted to be intrinsically disordered. It is necessary for the recruitment of the deadenylation machinery and the subsequent degradation of target mRNAs (26,27).

Roquin-1 was first discovered in a mouse germline mutagenesis screen for autoimmune regulators. The so-called sanroque mutation of the ROQ domain leads to the overexpression of its target *ICOS* (inducible T-cell costimulator), which causes the accumulation of follicular helper T cells and the production of autoantibodies (28,29). Further immune-relevant targets such as *Ox40* (*TNFRSF4*) and *A20* (*TNFAIP3*) were subsequently identified, underlining Roquin's role in the immune system (27,30). However, knockout of Roquin-1 or Roquin-2 causes perinatal lethality in mice, strongly suggesting additional roles of Roquin outside the immune system (30,31).

Roquin binding to so-called CDEs (constitutive decay elements) in the 3′UTR of target mRNAs has been best investigated so far. CDEs consist of a single hairpin that is capped with a tri-nucleotide loop. Mutational analysis of the CDE in the 3′UTR of *TNF* revealed the importance of a YRY triloop motif and three Y-R base pairs in the upper part of the stem forming a purine stack at the 3′ side (26). However, this consensus does not match CDE-like hairpins found in other Roquin target mRNAs (32,33). In addition, structural analyses of Roquin-1 or Roquin-2 in complexes with different CDEs showed mainly a shape-specific recognition independent of exact sequence requirements (33–36). Moreover, an ADE (alternative decay element) consisting of a hairpin capped with a U-rich hexaloop was identified as a functional, high-affinity Roquin binding site in the 3′UTR of *Ox40* (37). Together, these studies suggest that Roquin binding is much more versatile than was indicated by the mutational analysis of the *TNF* CDE. Thus, the exact sequence requirements for an active CDE are still unclear. However, exact knowledge of Roquin’s binding preference is necessary for the accurate genome-wide prediction of its targets.

Using a bioinformatic approach for the detection of conserved structures within UTRs, we identified two CDEs in the 3′UTR of *UCP3*. Performing an in-depth mutational analysis allowed us to define a new, relaxed consensus for active CDEs. Using this consensus, we identified a plethora of new Roquin targets. Additional targets encoding ADEs were also identified. Many of these new targets are not connected to immune responses, supporting Roquin’s role in other cellular processes. Quantification of target mRNA levels after Roquin knockdown showed, that several targets have cell-type-specific expression and were therefore not detected in previous studies.

## MATERIALS AND METHODS

### Cell culture

HEK293 cells (Leibniz-Institute DSMZ, Germany, ACC 305), C2C12 cells (Leibniz-Institute DSMZ, Germany, ACC 565) and HeLa ‘Flp-In Host Cell Line’ HF1-3 cells (38) were cultured in Dulbecco’s modified Eagle’s medium (DMEM, Sigma-Aldrich) supplemented with 10% (HEK293 and HeLa) or 20% (C2C12) fetal bovine serum (FBS Superior, Biochrom), 1 mM sodium pyruvate (Thermo Fischer Scientific) and Pen Strep (Thermo Fisher Scientific) at 37°C in a 5% CO_2_ humidified incubator. HF1-3 cells were additionally supplemented with 100 μg/ml zeocin (Invivogen). HF1-3 cells stably expressing *UCP3* wt or mutI/II constructs were supplemented with 150 μg/ml hygromycin B (Invivogen). HUVECs (Lonza) were cultured in EBM-Plus medium (Lonza) supplemented with EGM-Plus SingleQuots (Lonza) and 10% FBS (Thermo Fisher Scientific) at 37°C in a 5% CO_2_ humidified incubator. For differentiation experiments, C2C12 cells were grown to 90–100% confluency and medium was changed to DMEM (Sigma-Aldrich) supplemented with 2% horse serum (Sigma-Aldrich), 1 mM sodium pyruvate (Thermo Fischer Scientific) and Pen Strep (Thermo Fisher Scientific).

### Plasmid construction

#### pDLP (luciferase reporter system)

To generate luciferase reporter plasmids the 1131 bp long 3′UTR of *UCP3* (ENST00000314032.8) plus 98 nt endogenous context was introduced downstream of the firefly luciferase open reading frame into the multiple cloning site of pDLP (39) using NotI and HindIII restriction sites. The 3′UTR deletion mutant without the predicted structurally conserved window was generated by a two-step overlap extension polymerase chain reaction (PCR). All 100 nt long variants of the *UCP3* repressive element, other conserved CDEs or other Dynalign-predicted windows were generated by hybridization of complementary oligonucleotides. All sequences are summarized in Supplementary Table S1.

#### pHDV (*in vitro* transcription)

For RNA synthesis, UCP3 wt, MUTI, MUTII, MUTI/II, R1, CDEImut, CDEIImut, CDEI/IImut sequences together with the T7 promoter were generated by hybridization and introduced into the NcoI and HindIII sites of an HDV ribozyme encoding plasmid based on the pSP64 vector (Promega). RNAs were transcribed as HDV ribozyme fusions to obtain uniform 3′ ends. Required sequences were obtained by hybridization of complementary oligonucleotides and phosphorylation of restriction sites.

#### pCMV (miRNA expression)

For the overexpression of hsa-miR-152-5p, the genomic locus with 200 bp up- and downstream endogenous context was cloned in the XbaI and XmaI sites of the pCMV-MS plasmid (39).

#### pDF_FRT (stable integration)

For generation of the integration plasmid pDF_FRT, eGFP and mCherry expressed from a shared CMV enhancer/promoter element (pBI-CMV1; Clontech), were inserted into the NotI and NheI sites of the pcDNA5/FRT plasmid (Invitrogen). pDF_FRT_UCP3_wt and _MUTI/II were generated by hybridization of complementary oligonucleotides and insertion downstream of eGFP into the NotI and HindIII restriction sites of pDF_FRT.

The complete vector sequences are available upon request.

### Transient transfection

For transfection of reporter plasmids, 100 000 HEK293 cells were seeded in 24-well plates. Twenty-four hours after seeding, cells were transfected with 100 ng reporter plasmid using Lipofectamine 2000 (Thermo Fisher Scientific) according to the manufacturer’s protocol. Firefly and *Renilla* luciferase activity were measured 24 h post-transfection using the Dual Luciferase Reporter Assay System (Promega). For miR-152 overexpression, 400 ng pCMV-miR-152 or pCMV-MS and 100 ng reporter plasmid were cotransfected using Lipofectamine 2000.

For siRNA transfection, 100 000 or 200 000 HEK293 or HF1_3 cells were reverse transfected with 100 nM siRNA (siCTRL: 5′-UUCUCCGAACGUGUCACGU[dT][dT]-3′ or siROQ1/2 mix: 5′-CCAAGAAAUGUGUAGAAGA[dT][dT]-3′ and 5′-UCUUCUACACAUUUCUUGG [dT][dT]-3′) using Lipofectamine RNAiMAX (Thermo Fisher Scientific) according to the manufacturer’s protocol. RNA and protein samples were prepared 48 h post-transfection. To investigate the influence of Roquin knockdown on luciferase activity, transient transfection of the reporter plasmid was performed 24 h after reverse transfection of siRNAs. For siRNA transfection, 350 000 HUVECs were seeded in 60 mm plates. 24 h after seeding, cells were transfected using Lipofectamine RNAiMAX (Thermo Fisher Scientific) and 100 nM siRNA (siCTRL or siROQ1/2 mix). RNA and protein samples were prepared 48 h post-transfection. For siRNA transfection, 50 000 of C2C12 cells were reverse transfected with 100 nM siRNA (siCTRL or mmsiROQ1/2 mix: 5′-CGCACAGTTACAGAGCTCA[dT][dT]-3′ and 5′-GGACTTGGCTCATAAATCA[dT][dT]-3′) using Lipofectamine RNAiMAX according to the manufacturer's protocol. Forty-eight hours post-transfection cells had reached 90–100% confluency and differentiation was induced by switching to culture media containing 2% horse serum. Twenty-four hours post-induction of differentiation RNA and protein samples were prepared.

### Genomic integration

For the establishment of constitutive expression of GFP with the UCP3 wt repressive element and the double mutant MUTI/II, the Flp-In System (Thermo Fisher Scientific) was used. The pFRT-constructs were cotransfected with pOG44 (Thermo Fisher Scientific) in a molar ration 1:9 into HF1-3 cells using Lipofectamine 2000 (Thermo Fisher Scientific). Two days after transfection, cells were cultivated in selection medium supplemented with 150 μg/ml hygromycin B. The selection steps were as described in the manufacturer’s protocol. After 2 weeks of cultivating the cells with 150 μg/ml hygromycin B genomic integration was analyzed by flow cytometry.

### Flow cytometry

For flow cytometry, cells were washed with Phosphate-buffered saline (PBS) once and detached by Trypsin digestion. After Trypsin treatment cells were diluted 1:1 with PBS and fluorescence at 510 and 610 nm was measured with a Beckman Coulter Cytoflex S.

### RNA extraction, cDNA synthesis and qPCR

Total RNA from HeLa, C2C12 and HEK293 cells was extracted with TRIzol Reagent (Ambion) followed by Turbo DNase (Thermo Fisher Scientific) treatment. Total RNA from HUVECs was isolated using the miRNeasy Mini kit (Quiagen), including on-column DNA digestion with the RNase-Free DNase Set (Qiagen). After isolation, 1 μg of RNA was quality checked on a 1% agarose gel.

MiRNAs were quantified according to (40). For mRNA analysis, total RNA was reverse transcribed using MuLV reverse transcriptase (Thermo Fisher Scientific) and quantified via qPCR using the Fast SYBR Green Master Mix (Thermo Fisher Scientific). For details see Supplementary Methods.

Oligonucleotides used for cDNA synthesis and qPCR are listed in Supplementary Table S2.

### mRNA decay assay

For mRNA decay experiments, HF1-3 cells stably expressing either *UCP3* wt or *UCP3* MUTI/II were treated with 5 mg/ml actinomycin D (Sigma). Total RNA was extracted at the indicated time points and *GFP* mRNA levels quantified by RT-qPCR (oligonucleotides are listed in Supplementary Table S2). *GFP* mRNA levels were normalized to *RPLP0* mRNA levels and plotted against time. MRNA half-lives were calculated as follows: Y = (Y0 - Plateau)*e ^(-K*X) + Plateau^

### *In vitro* transcription

pHDV constructs were linearized with HindIII and purified by phenol extraction. A total of 2 μg of linearized DNA were transcribed overnight at 37°C using the following conditions: 200 mM Tris–HCl pH 8.0, 20 mM Mg(Ac)_2_, 50 mM Dithiothreitol (DTT), 2 mM spermidine, 4 mM nucleoside triphosphate (NTP) (each) and 3 μg T7 Polymerase (homemade). RNA was polyacrylamide gel electrophoresis (PAGE) purified.

### In-line probing

In-line probing was performed according to (41). For details see Supplementary Methods.

### Electromobility shift assays (EMSAs)

Electromobility shift assays (EMSAs) were performed according to (33). For details see Supplementary Methods.

### RNA affinity purification

Purified RNA was dephosphorylated using calf intestine phosphatase (Roche). For 5′ end biotinylation, 600 pmol RNA were incubated with 0.2 mM γ-S-ATP (Biomol) and T4 polynucleotide kinase (New England Biolabs) for 30 min at 37°C. Biotin-long-arm maleimide (Vector Laboratories) was added and incubated for 30 min at 65°C. Unincorporated label was depleted by LiCl precipitation. Biotinylated RNA (200 pmol) was conjugated to Dynabeads M-280 (Invitrogen) in incubation buffer (10 mM Tris–HCl pH 7.4, 150 mM KCl, 0.5 mM DTT, 0.05% NP40, 100 U/ml RNasin) for 2 h at 4°C with continuous rotation. Whole cell protein lysate (1 mg) of HEK293 cells together with 200 μg yeast tRNA (Sigma-Aldrich) and 5 mg Heparin (Sigma-Aldrich) was added to the beads and incubated for 1 h at 4°C followed by 15 min at room temperature with continuous rotation. Beads were washed five times with incubation buffer, resuspended in 30 μl protein loading dye and boiled at 95°C for 10 min. Eluted proteins were separated by sodium dodecyl sulphate-polyacrylamide gel electrophoresis (BioRad).

### Mass spectrometry

Mass spectrometric analysis of RNA bound proteins were performed basically as described at (42) with the following changes: (i) samples were run on NuPAGE gel and entire lanes were cut into 23 slices and further processed as described (see above). (ii) LC-MS analyses were performed on a Q-Exactive Plus MS (Thermo Fisher Scientific) coupled to an UltiMate 3000 HPLC (Thermo Fisher Scientific) under similar condition as described (see above) except that LC separation time was 48 min. The MS data were acquired by scanning the precursors in mass range from 350 to 1600 m/z at a resolution of 70 000 at m/z 200. Top 20 precursor ion were chosen for MS2 by using data-dependent acquisition mode at a resolution of 15 000 at m/z 200 with maximum IT of 50 ms. (iii) Data analysis was performed as described (see above) using Mascot (Version 2.3.02) as search engine searching against Uniprot database (Taxonomy human, 155 190 entries, 20161124). Data were evaluated with Scaffold software (Version 4.8.2).

### Western blot

Cells were lysed in lysis buffer [137 mM NaCl, 10% glycerol, 20 mM Tris–HCl pH 8.0, 2 mM ethylenediaminetetraacetic acid pH 8.0, 1% Igepal, 5 μl protease inhibitor cocktail (Sigma) for 20 min on ice. After centrifugation (15 min at 17 000 *g*, 4°C) the protein content in the soluble fraction was determined according to the Bradford method. A total of 10–20 μg protein were loaded onto precast gels with stain free technology (Bio-Rad). Total lane protein was visualized as loading control with the ChemiDoc Imaging System (Bio-Rad) prior transfer onto PVDF membranes (Bio-Rad). The primary antibody Roquin-1/-2 (Millipore 3F12) and the secondary antibody Horseradish peroxidase-conjugated anti-rat IgG (Jackson Immunoresearch) were used. Blots were developed with the ECL select (Life technologies). Imaging was performed with the ChemiDoc Imaging System and quantified using Image Lab Software (Bio-Rad).

### Computational methods

A detailed description of (i) the search for conserved structures by Dynalign, (ii) the prediction of human CDEs and ADEs and their folding probability and (iii) the evolutionary conservation of putative CDEs and ADEs is provided in the Supplementary Methods.

### Statistical analysis

All bar graphs are given as mean values ± standard deviation. Statistical analysis was done using Student’s *t*-test (two-tailed, paired).

## RESULTS

### A conserved structure in the 3′UTR of *UCP3* reduces gene expression

In order to identify functional structured mRNA elements, we investigated the conservation of structures on a genome-wide scale, by comparing all UTRs of human and mouse. Besides the human genome, the mouse genome is currently the best annotated and was therefore selected for comparison. Further emphasis was placed to rely on structural conservation. Therefore, the bioinformatic prediction of conserved structures was performed using the RNA folding program Dynalign, which simultaneously folds and aligns two input sequences (23,43). It predicts the conserved secondary structure with the lowest total score, which is the sum of the folding free energies of the two structures as estimated by nearest neighbor rules and a score for the alignment. The alignment is made to reflect the conserved structure, and this alignment does not consider sequence identity. Therefore, the accuracy of Dynalign is not adversely affected when sequence identity is low (21,43). This is a strong advantage compared to programs that predict RNA secondary structures based on a fixed sequence alignment. These depend on the quality of the sequence alignment and as a result favor higher sequence similarities (44). Therefore, Dynalign includes the advantage of evolutionary conservation for structure prediction, without the disadvantage of relying on high-sequence conservation.

For the discovery of structured RNA sequences in genomes, machine learning was used to train a model to classify Dynalign output (21,45) (see also http://hdl.handle.net/1802/6609). Two sequences are input to Dynalign, the best scoring common structure is predicted, and features of the sequences (length and nucleotide composition) and the estimated folding free energy change are used to estimate the probability that the two sequences represent a conserved and stable RNA secondary structure. For the genome-wide search, all human UTRs were divided into 100 nt long windows, with a step size of 50 nt and structurally compared to the corresponding mouse sequences. A total of 100 nt long windows were chosen, to balance structure prediction accuracy and scanning efficiency. The predicted structurally conserved windows are given in Supplementary File 1.

We chose 20 windows from 3′UTRs for an initial testing of post-transcriptional regulation (Supplementary Table S3). The windows were selected based on an estimated probability of >0.9 to be a conserved structure, a total 3′UTR length between 500 and 1500 nt and they were not allowed to overlap with other windows. The 100 nt long 3′UTR sequences were cloned downstream of a luciferase reporter gene. Four of the 20 windows significantly reduced luciferase activity in HEK293 cells (Supplementary Figure S1). The most efficient reduction was observed with the window from the 3′UTR of *UCP3*. The *UCP3* gene codes for a mitochondrial membrane protein. It is implicated in fatty acid metabolism, obesity and insulin resistance [reviewed in (46,47)].

**Figure 1.**
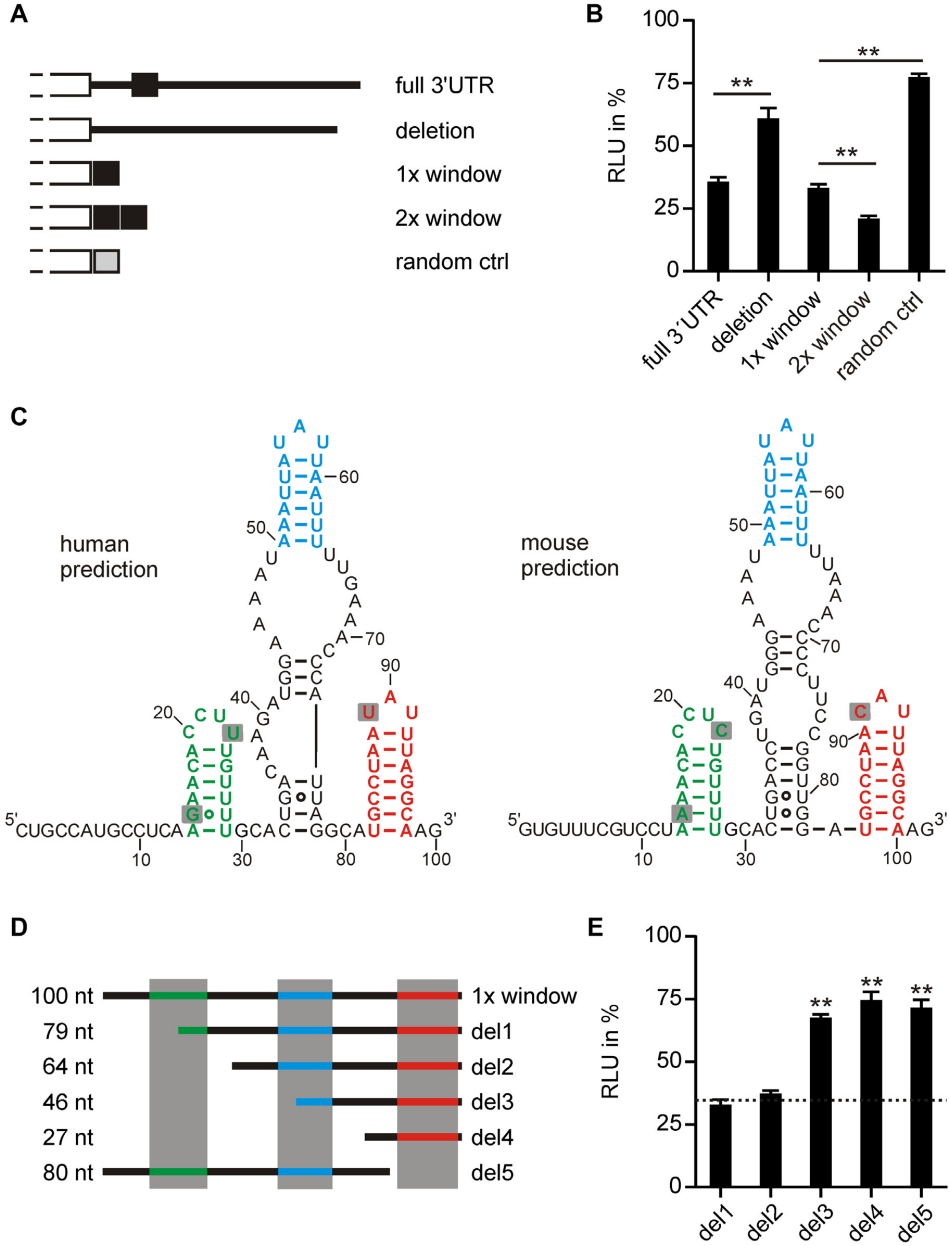
The 100 nt long structurally conserved region in the 3′UTR of *UCP3* codes for a repressive element. (**A**) Overview of luciferase reporter constructs. Different fragments of the *UCP3* 3′UTR were fused to firefly luciferase. (**B**) Luciferase activity of *UCP3* 3′UTR fusion constructs shown in (A). Firefly luciferase activity was normalized to *Renilla* luciferase as internal transfection control. Values are normalized to an empty vector control, without *UCP3* 3′UTR sequences. *n* = 3. (**C**) Secondary structure prediction by Dynalign of the structurally conserved regions in the human and mouse *UCP3* 3′UTRs. (**E**) Overview of truncations of the 100 nt long window. (**D**) Luciferase activity of *UCP3* truncation constructs shown in (E). Firefly luciferase activity was normalized to *Renilla* luciferase as internal transfection control. Values are normalized to an empty vector control, without *UCP3* 3′UTR sequences. Luciferase activity of the 100 nt window is indicated as dashed line. *n* = 3. (**) *P*-value < 0.01.

To further confirm the importance of this element in *UCP3* expression, we fused the complete 3′UTR of *UCP3* (1131 nt) and a variant where the 100 nt window is deleted to luciferase (Figure 1A and B). The complete 3′UTR and the window alone showed the same level of luciferase repression, while deleting the 100 nt window restored luciferase activity. Doubling of the window further reduced luciferase activity, while a randomized control with the same nucleotide composition again restored luciferase activity (Figure 1A and B). Thus, the 100 nt structurally conserved region identified by Dynalign is necessary and sufficient for post-transcriptional regulation by the *UCP3* 3′UTR.

Examination of the structurally conserved region for miRNA binding sites using the TargetScan program (48) revealed two conserved, overlapping sites that are targeted by two miRNA families, miR-148a/148b/152 and miR-130a/130b/301a/301b (Supplementary Figure S2A and B). All seven of these miRNAs are either undetectable or expressed at a very low level in HEK293 cells (Supplementary Table S4). Thus, it is unlikely that they are responsible for the observed reduction in luciferase activity. Accordingly, deletion of the binding sites had no effect on luciferase repression (Supplementary Figure S2C). To test, if the predicted miRNA binding sites are functional at all, we overexpressed one of the miRNAs, miR-152, in HEK293 cells. Overexpression of miR-152 reduced luciferase activity of the wild-type (wt) sequence, but not that of the deletion construct (Supplementary Figure S2C and D). Thus, although these miRNAs are not responsible for the reduction in luciferase activity observed in HEK293 cells, they might control UCP3 levels in other cell types.

The conserved structure of the *UCP3* element predicted by Dynalign contains three small hairpins separated by sequences that are less conserved at both structure and sequence level (Figure 1C). To assess the importance of the three hairpins, we performed truncation studies of the *UCP3* element (Figure 1D and E). Deleting the first hairpin alone (del1) or together with the miRNA binding site (del2) had no effect. However, deleting the second (del3 and del4) or third hairpin (del5) restored luciferase activity. The minimal active construct del2 is very AU-rich (72% AU content). To exclude unspecific luciferase reduction due to this high AU content, we fused five different randomized sequences with the same nucleotide composition (R1–R5) and one randomized sequence with uniform nucleotide content (64 nt) to the luciferase reporter. All randomizations restored luciferase activity (Supplementary Figure S3). This confirms that a minimal region of 64 nt is necessary and sufficient to regulate gene expression. The del2 construct will be referred to as *UCP3* wt henceforth.

**Figure 2.**
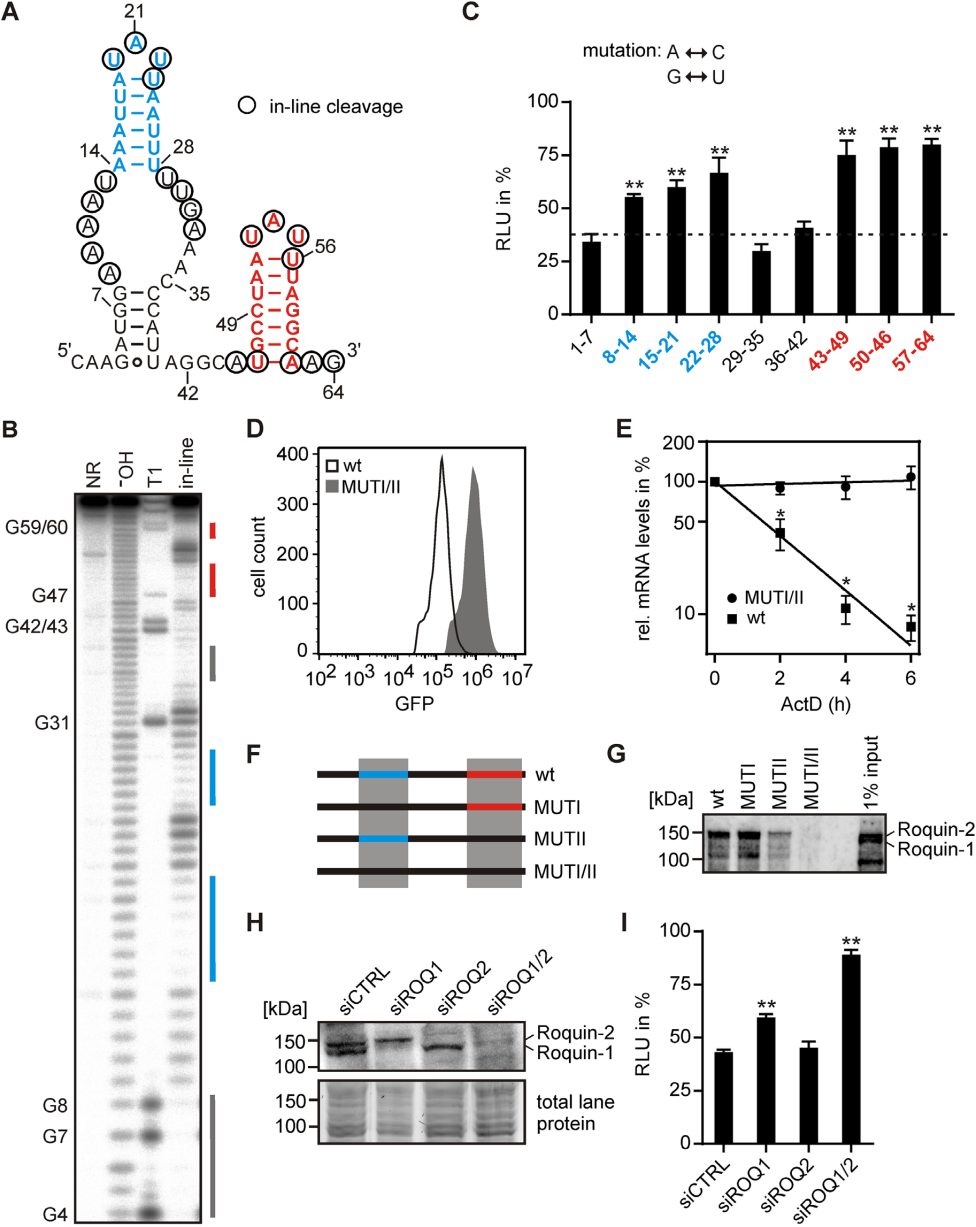
The *UCP3* wt element folds into two hairpins and reduces mRNA half-life by interaction with Roquin. (**A**) Predicted lowest free energy secondary structure of the *UCP3* wt element by RNAstructure. Nucleotides detected by in-line probing [shown in (**B**)] are circled. (**B**) In-line probing analysis of *UCP3* wt RNA. The RNA was loaded directly (NR, no reaction), subjected to cleavage by RNase T1 or alkaline hydrolysis (**_¯_**OH), or incubated for 40 h at room temperature and pH 8.3 (in-line) prior to Urea PAGE. Paired regions are indicated by identically colored lines. (**C**) Luciferase activity of *UCP3* mutants for the identification of motifs essential for gene regulation. Adenine was mutated to cytosine, guanine to uracil and *vice versa*. Numbers indicate mutated nucleotide positions. Firefly luciferase activity was normalized to *Renilla* luciferase as internal transfection control. Values are normalized to an empty vector control, without *UCP3* 3′UTR sequences. Luciferase activity of the *UCP3* wt element is indicated as dashed line. *n* = 3. (**D**) GFP fluorescence of *UCP3* wt and double mutant (MUTI/II). GFP-UCP3-fusion constructs were stably integrated into the genome of HeLa cells. GFP fluorescence was measured by flow cytometry. (**E**) Half-life of *GFP* mRNAs containing the *UCP3* wt element (wt) or double mutant (MUTI/II). HeLa cells stably expressing one of the two constructs were treated with 5 μg/μl actinomycin D (ActD). Thereafter, total RNA was isolated at 2 h intervals and *GFP* mRNA levels quantified by RT-qPCR. *GFP* values are normalized to the housekeeping gene *RPLP0. n* = 3. (**F**) Overview of *UCP3* constructs used for RNA affinity purification. (**G**) Analysis of Roquin binding to the *UCP3* constructs shown in (**F**). For RNA affinity purification HEK293 whole cell lysates were incubated with the different *UCP3* RNAs. Roquin-1 and Roquin-2 were visualized by western blot using anti-Roquin antibody. *n* = 2. (**H**) Western blot of Roquin-1 and Roquin-2 after siRNA-mediated knockdown. Anti-Roquin was used to verify the respective knockdown. Total lane protein is shown as loading control. *n* = 3. (**I**) Luciferase activity of the *UCP3* wt element after siRNA-mediated knockdown of Roquin proteins. Firefly luciferase activity was normalized to *Renilla* luciferase as internal transfection control. Values are normalized to an empty vector control, without *UCP3* 3′UTR sequences. *n* = 3. (**) *P*-value < 0.01. (*) *P*-value < 0.05.

We performed in-line probing to verify the structure prediction of the *UCP3* wt element. In-line probing of the 64 nt long region confirmed the existence of the two hairpins (Figure 2A and B). The basal A-U bp of the second hairpin is not formed *in vitro*, so that both hairpins have a 6 bp stem. Furthermore, a small stem of 5 bp is formed by the non-conserved surrounding sequences. In order to precisely map the regions important for post-transcriptional regulation, we performed a mutational analysis of the 64 nt in blocks of 7 nt. Mutations that interfered with the formation of one of the two hairpins restored luciferase activity, while mutations affecting only the surrounding sequences, including the small 5 bp stem, had no effect (Figure 2C). Of note, mutation of nucleotides 8–14, which shortens the stem of the first hairpin by 1 bp down to 5 bp, already significantly increased luciferase activity.

To determine whether the *UCP3* wt element reduces gene expression by reducing mRNA abundance or translation efficiency, we determined the half-lives of mRNAs encoding either the *UCP3* wt element (wt) or a double mutant in which formation of both hairpins is prevented (MUTI/II). The *UCP3* wt and double mutant were fused to a GFP reporter gene and stably integrated into the genome of HeLa cells. Successful integration was monitored by the simultaneous integration of an mCherry reporter gene (Supplementary Figure S4A). Measurement of GFP levels by flow cytometry showed the expected reduction (7.7-fold) of GFP levels by the *UCP3* wt element compared to the double mutant (Figure 2D). A corresponding reduction was observed at mRNA level (Supplementary Figure S4B). Determination of mRNA half-lives after treatment with actinomycin D, which blocks transcription, showed that *GFP* mRNA containing the *UCP3* wt element in its 3′UTR is very unstable (half-life of ∼1.5 h) compared to the double mutant, for which no reduction of mRNA levels was detectable over the time course of the experiment (Figure 2E).

In summary, we have identified a *cis*-acting repressive element in the 3′UTR of *UCP3* coding for two hairpins with a 6 bp long stem by bioinformatic prediction of conserved structures.

### The 3′UTR of *UCP3* encodes two Roquin binding sites

Next, we performed RNA affinity purification to identify protein factors involved in the post-transcriptional regulation of *UCP3*. HEK293 whole cell lysate was incubated with biotinylated RNAs, of either the wt *UCP3* element (wt), individual mutations of the first or second hairpin (mutI or mutII, respectively), a double mutant (mutI/II) and a randomized control with the same nucleotide composition (R1) (Supplementary Figure S5). A total of 229 proteins were detected by mass spectrometry using a cut-off of at least five unique peptides detected in at least one of the samples. Ten of these proteins were not detected in the two controls, i.e. the double mutant and the randomized sequence (Supplementary Table S5). Among these 10, the paralogs Roquin-1 and Roquin-2 were highly enriched. The binding of Roquin-1 and Roquin-2 to RNAs, which form at least one of the two hairpins, but not to the double mutant, was confirmed by western blot analysis after RNA affinity purification (Figure 2F and G).

To determine whether Roquin mediates repression by binding to the *UCP3* wt element, we measured luciferase activity after transient knockdown of Roquin-1, Roquin-2 or both. In accordance with the previously reported redundant function of Roquin-1 and Roquin-2 in the repression of gene expression, only the double knockdown fully restored luciferase activity in HEK293 cells (Figure 2H and I). In addition, the double knockdown of Roquin-1 and Roquin-2 increased GFP levels in HeLa cells, which stably express *GFP* mRNA containing the *UCP3* wt element in its 3′UTR (Supplementary Figure S6). *UCP3* is mainly expressed in the skeletal muscle. Thus, we performed a double knockdown of Roquin-1 and Roquin-2 in C2C12 mouse myoblasts, to verify the regulation of endogenous *UCP3* by Roquin (Supplementary Figure S7A and B). After 1 day of differentiation, *UCP3* mRNA levels were ∼3-fold higher in Roquin knockdown cells compared to control cells (Supplementary Figure S7C). These experiments confirm Roquin’s role in the repression mediated by the *UCP3* 3′UTR element.

Together, mutational analysis, structural probing and RNA affinity purification suggested binding of Roquin to the two hairpins. These are similar to CDEs in that they form a 6 bp long stem capped by a tri-nucleotide loop and are referred to as CDEI and CDEII henceforth.

**Figure 3.**
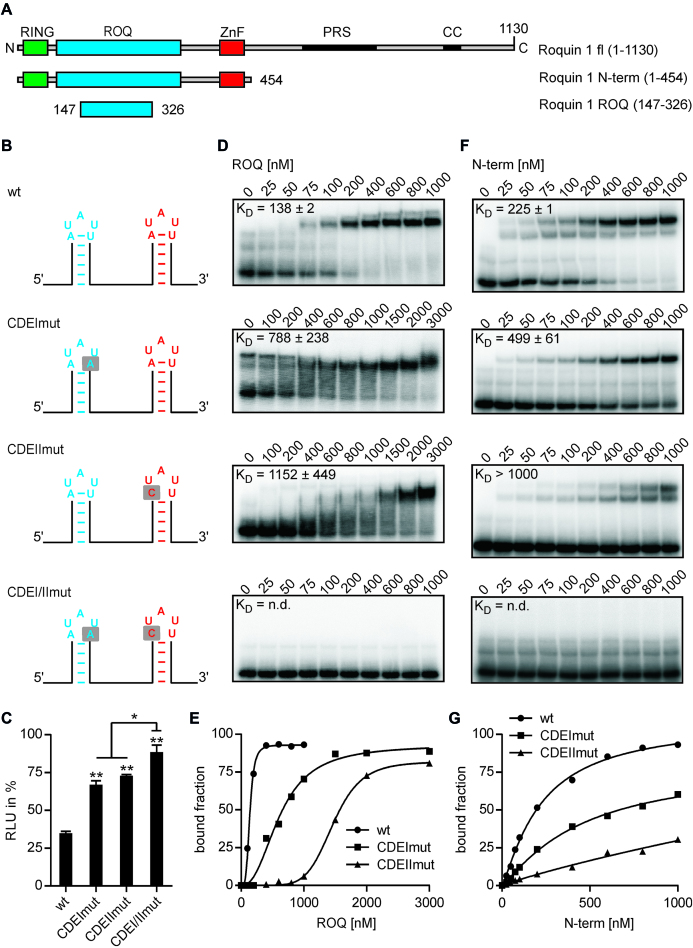
Both *UCP3* CDEs are required for efficient Roquin binding. (**A**) Domain organization of mouse Roquin-1 and overview of Roquin fragments used for binding experiments. (**B**) Overview of *UCP3* constructs used for binding experiments. (**C**) Luciferase activity of *UCP3* constructs shown in (B). Firefly luciferase activity was normalized to *Renilla* luciferase as internal transfection control. Values are normalized to an empty vector control, without *UCP3* 3′UTR sequences. *n* = 3. (**D** and **F**) Binding of recombinant Roquin-1 to the *UCP3* constructs shown in (B). Radiolabeled RNAs were incubated with increasing amounts of Roquin-1 ROQ domain (D) or N-terminus (F). The apparent dissociation constant (*K*_D_) was calculated from two to three independent experiments. (**E** and **G**) Representative quantification of EMSA experiments with Roquin-1 ROQ domain (E) or N-terminus (G). (**) *P*-value < 0.01. (*) *P*-value < 0.05. n.d. = not determined.

However, while RNA affinity purification suggested an independent binding of Roquin to both CDEs, mutation of one of the two CDEs alone is sufficient to restore luciferase activity. To investigate this discrepancy more closely, we performed electrophoretic mobility shift analyses (EMSAs) with purified Roquin-1 protein and the *UCP3* wt element or single and double mutants of the two CDEs (Figure 3A). To minimize the effects on the overall folding of the *UCP3* element, we mutated only 1 nt of the closing base pair so that triloop formation is prevented (Figure 3B and Supplementary Figure S8). In accordance with our previous results, mutation of one of the two CDEs into a pentaloop is sufficient to restore luciferase activity to 67–73% of the empty vector control (CDEImut and CDEIImut) (Figure 3C). The combined mutation of both CDEs further increased luciferase activity to 89% of the empty vector control (CDEI/IImut). This small, but significant increase, suggests that Roquin can bind to both hairpins independently, but also that both CDEs are necessary for robust regulation of gene expression. This finding is confirmed by EMSAs with Roquin and the various *UCP3* mutants. Both the core ROQ domain alone and the complete N-terminus showed a higher affinity to the wt than to the two single mutants (Figure 3D–G). No binding to the double mutant could be observed (Figure 3D and F). Binding the N-terminal Roquin fragment to the *UCP3* wt generated two bands, which is consistent with two N-terminal fragments binding to the two CDEs. However, binding of the core ROQ domain to the *UCP3* wt resulted in only one band. This migrated slower than the bands observed with the two single mutants, suggesting the binding of two core ROQ domains to the *UCP3* wt. We performed a stoichiometric binding experiment (49) to verify that actually two core ROQ domains are bound to the *UCP3* wt. It confirmed that two core ROQ domains bind to the *UCP3* wt (Supplementary Figure S9A), consistent with the simultaneous detection of both CDEs. Roquin binding to these two CDEs is mediated by its core ROQ domain, as the mutation of this domain, which has been shown to inhibit binding to the *TNF* CDE, also inhibits binding to the *UCP3* CDEs (Supplementary Figure S9B).

The observed *K*_D_ of 138 nM of the core ROQ domain of Roquin-1 to the *UCP3* wt is comparable to other high-affinity binding sites, such as the *TNF* CDE (26,33,50), the *ICOS* CDE (51) and the *Ox40* ADE (37). In contrast, the ROQ domain of Roquin-1 showed a significantly lower affinity to the *Ox40* CDE, with a *K*_D_ > 1000 nM comparable to CDEIImut (37). For the ROQ domain of Roquin-2 a *K*_D_ of ∼ 360 nM to the *Ier*3 CDE was measured (36). Thus, the binding constants of the wt and the single mutants are in the range of previously determined binding constants of Roquin to CDEs and ADEs. Recently, the binding of an N-terminal fragment to a tandem element from the *ICOS* 3′UTR, encoding a CDE-like and an octaloop-containing stem loop, was tested by EMSAs (32). As was the case in our studies, efficient binding was only observed if both stem loops were present.

In summary, the *UCP3* 3′UTR encodes two CDEs, both necessary for efficient Roquin binding and robust suppression of gene expression.

### Mutation analysis of the *UCP3* CDEs shows the versatility of Roquin binding

**Figure 4.**
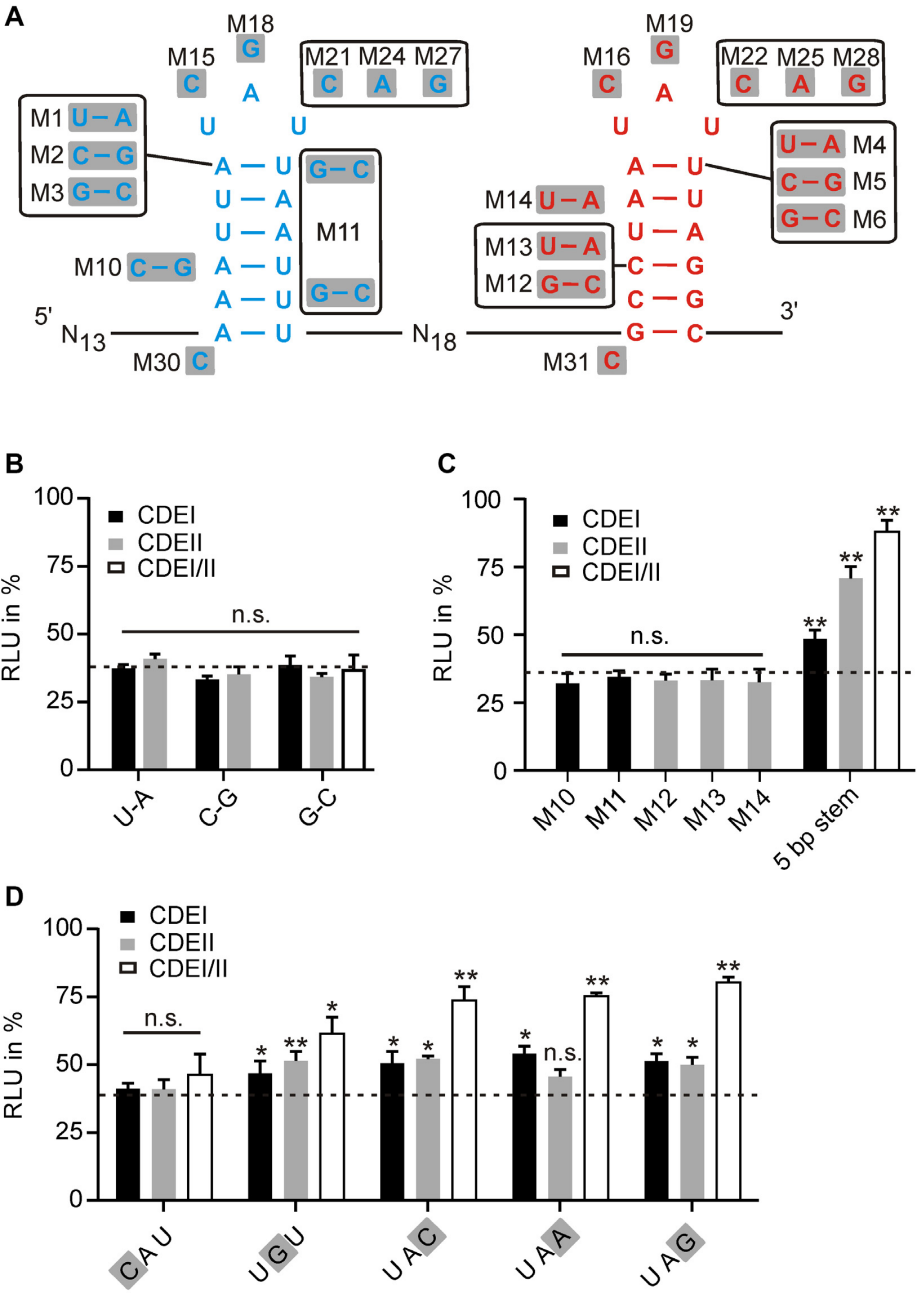
Mutational analysis of the *UCP3* tandem CDE. (**A**) Overview of *UCP3* mutants. (**B**) Luciferase activity of closing base pair mutants. *n* = 3. (**C**) Luciferase activity of stem mutants. *n* = 3. (**D**) Luciferase activity of triloop mutants. *n* = 3. (B–D) Firefly luciferase activity was normalized to *Renilla* luciferase as internal transfection control. Values are normalized to an empty vector control, without *UCP3* 3′UTR sequences. Luciferase activity of the *UCP3* wt element is indicated as dashed line. (**) *P*-value < 0.01. (*) *P*-value < 0.05. n.s. = not significant.

The CDEs found in the *UCP3* 3′UTR deviate from the consensus derived by mutational analysis of the *TNF* CDE. They do not code for three Y-R base pairs in the apical part of the stem, nor does CDEI contain a purine stack at the 3′ side. This is in line with structural analyses that did not show sequence-specific contacts to the stem region upon Roquin binding to CDEs (33–36). To evaluate the contribution of the stem sequence to gene regulation, we performed a comprehensive mutational analysis of the two CDEs of *UCP3* (Figure 4A). Mutation of the base pair that closes the tri-nucleotide loop to any other Watson–Crick base pair did not affect luciferase repression (Figure 4B). Mutation of the closing base pair to a G$\circ$U or U$\circ$G wobble base pair significantly increased luciferase activity, but not to the same extent as the pentaloop mutation, suggesting that wobble base pairs may also occur in endogenous Roquin binding sites (Supplementary Figure S10). Stabilization of CDEI, which consists exclusively of A-U base pairs, by one or two G-C base pairs, had no effect on repression. Similarly, neither the stabilization nor destabilization of the CDEII stem changed luciferase activity (Figure 4B and C). In addition, the complete exchange of CDEI with CDEII also had no effect (Supplementary Figure S10). This indicates that stem (in-)stability as such is not an important feature for recognition by Roquin. However, stem length seems to be important. Mutational analysis of the *TNF* CDE showed that extending the stem to 9 bp inactivates the CDE (26). Conversely, the mutagenesis screen of the *UCP3* wt indicates that shortening the stem to 5 bp significantly impairs repression (Figure 2C). To verify the dependence on stem length, we introduced point mutations to shorten the stem of CDEI, CDEII or both to 5 bp. Such shortening significantly restored luciferase activity and confirms that CDEs with only 5 bp long stems are less active (Figure 4C). Furthermore, neither the introduction of a purine stack in CDEI (M1) nor the destruction of the purine stack in CDEII (M12) influenced gene repression (Figure 4B and C). From this analysis, we conclude that a purine stack on the 3′ side of the stem is not a prerequisite for Roquin binding.

Further, we investigated the importance of the triloop sequence for gene regulation. Previous *in vitro* studies suggested YRN as a possible loop motif (25), but this was not comprehensively tested *in vivo*. Thus, we mutated nucleotides 1–3 to the corresponding other possible nucleotides in CDEI, CDEII or both and measured the luciferase activity of the mutants (Figure 4D). The exchange from U to C in position 1 had no influence on gene repression, in accordance with previous studies. However, mutation of A to G in position 2 significantly increased luciferase activity, both in CDEI, CDEII and the double mutant. This is in contrast to the *TNF* CDE, where A and G are equally effective in gene repression. The mutation of U at position 3 to any other nucleotide increased luciferase activity, which is particularly pronounced in double mutants. From this we conclude that YAU triloops are most efficient for gene regulation. This corresponds to the conservation of *UCP3* CDEs in mammals, which almost exclusively show YAU triloops (Supplementary Figure S11). However, other sequences that correspond to the YRN motif retain a certain regulatory capacity and can therefore occur in endogenous Roquin binding sites.

In summary, Roquin has no sequence preferences within the stem region and only minimal sequence requirements for the triloop. This finding is in line with the structural analyses of the Roquin CDE complexes, which suggested an almost exclusive shape-specific recognition (33–36). Further, it suggests that many more genes could encode functional CDEs and thus be regulated directly by Roquin.

### Identification of new Roquin target genes

**Figure 5.**
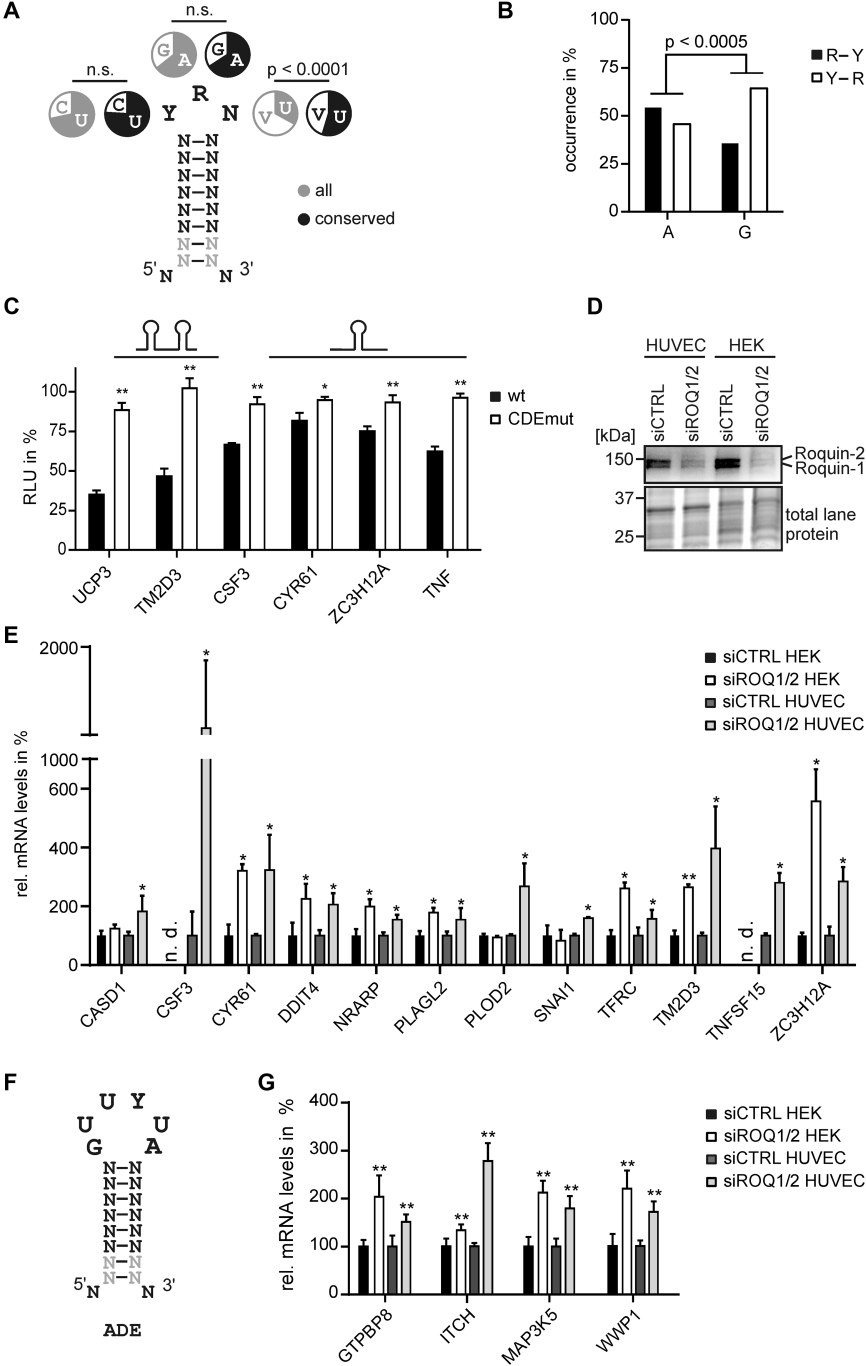
Identification of new Roquin targets. (**A**) Consensus used for bioinformatic prediction of new CDE structures by RNAMotif. Nucleotide occurrences in the triloop are shown for all and conserved elements. V = A, G, C. *P*-value < 0.0001 (Pearson’s chi-square test). (**B**) Co-occurrence of A or G at position 2 in the triloop with R-Y or Y-R closing base pairs. R = A, G and Y = C, U. *P*-value < 0.0005 (Pearson’s chi-square test). (**C**) Luciferase activity of different CDE-encoding 3′UTR regions and mutants. *n* = 3. CDEs and mutations are shown in Supplementary Figure S13. Firefly luciferase activity was normalized to *Renilla* luciferase as internal transfection control. Values are normalized to an empty vector control, without *UCP3* 3′UTR sequences. Influence of predicted CDE-like elements on luciferase activity in HEK293 cells. *n* = 3. (**D**) Western blot of Roquin-1 and Roquin-2 after siRNA-mediated knockdown in HEK293 cells and HUVECs. Anti-Roquin was used to verify the respective knockdown. Total lane protein is shown as loading control. *n* = 3. (**E**) RT-qPCR quantification of new Roquin target genes encoding CDEs after siRNA-mediated knockdown of Roquin-1 and Roquin-2 in HEK293 cells and HUVECs. Values are normalized to the housekeeping gene *RPLP0. n* = 4. (**F**) Consensus used for bioinformatic prediction of new ADE structures by RNAMotif. (**G**) RT-qPCR quantification of new Roquin target genes encoding ADEs after siRNA-mediated knockdown of Roquin-1 and Roquin-2 in HEK293 cells and HUVECs. Values are normalized to the housekeeping gene *RPLP0. n* = 4. (**) *P*-value < 0.01. (*) *P*-value < 0.05. n.d. = not detected.

Based on our mutational analysis of the *UCP3* CDEs, we created a new, relaxed consensus for the genome-wide prediction of functional CDEs in all human 3′UTRs (Figure 5A). The consensus consists of a 6–8 bp long stem capped with a YRN tri-nucleotide loop. We chose this stem length because our mutational analysis showed a significant reduction in repressive strength already for a 5 bp stem (Figure 4C) and mutation of the *TNF* CDE that a 9 bp long stem is not functional at all. Furthermore, we have excluded G°U wobble base pairs for this search, as this is the only stem mutation that significantly reduced the repressive activity of the *UCP3* CDEs (Supplementary Figure S10).

Scanning all human 3′UTRs showed that ∼2600 genes contain at least one potential CDE (Supplementary File 2). Because correct folding is crucial for the function of CDEs, we have calculated the folding probability of all predicted CDEs. These calculations used an exact calculation of the estimated thermodynamics and expanded on prior work that calculated loop probabilities (see Supplementary Methods) (52). Known CDEs, e.g. from the *TNF, Ox40* and *ICOS* mRNAs, have a folding probability between 15 and 98%. CDEII of *UCP3* is in the middle with 55%, while CDEI, which consists entirely of A-U base pairs, is significantly lower with 6% folding probability. About a third of the predicted CDEs have an even lower folding probability of <5% and might thus not be targeted by Roquin.

In addition, we studied the evolutionary conservation of all predicted potential CDEs as indication of their functional importance. The predicted human CDEs were queried in Multiz 46-way multiple sequence alignments with chimpanzee, mouse, dog and cow. A CDE is considered conserved either if its nucleotide sequence is identical across all five species or if it contains only nucleotide changes that still retain the structure of the stem and correspond to the description of the triloop. These tolerated changes include: (i) changes in the triloop that still fit the definition of the motif (5′ stem—YRN—stem 3′), (ii) changes in the length of the stem such that only 6 bp need to be conserved, and changes in the stem that can still form canonical base pairs. These can be (iii) compensating base pair changes or (d) G°U wobble base pairs.

Approximately one out of eight of the potential human CDEs is also conserved in the mammals studied, underlining their functional importance (Supplementary Table S6). Conserved CDEs show a pronounced enrichment for U at position 3 in the triloop, while the nucleotide distribution at positions 1 and 2 is identical between conserved and non-conserved CDEs (Figure 5A). This is consistent with our mutational analysis, which has shown that CDEs with U at this position show the strongest repression. In addition, conserved CDEs also have a significantly higher estimated likelihood of folding (Supplementary Figure S12). We also investigated preferences in the composition of the stem. Because the stem of CDEI in *UCP3* consists exclusively of A-U base pairs, we have calculated the AU content for all CDEs. There is no significant enrichment for AU-rich stems (>50%) in conserved versus non-conserved CDEs (Supplementary Figure S12). We further analyzed the presence of purine stacks (at least three consecutive purines) on the 3′ side of the stem. While the mutational analysis of the *TNF* CDE indicated the necessity of a purine stack at the 3′ side, our mutational analysis could not confirm this. In agreement, conserved CDEs have only a slight preference over non-conserved CDEs for a purine stack at the 3′ side (Supplementary Figure S12). Thus, purine stacks are found in CDEs, but are not required for their activity.

Our mutational analysis of the *UCP3* tandem CDE showed that G in position 2 of the triloop is less efficient in gene repression than A, while A and G are equally effective in the *TNF* CDE. One difference between the CDEs of the two genes is the polarity of the closing base pair. While all closing base pairs are equally effective in the context of the *UCP3* tandem CDE, a preference for Y-R closing base pairs was shown for the *TNF* CDE. Thus, we analyzed the co-occurrence of A or G at position 2 in the triloop with either Y-R or R-Y closing base pairs in conserved CDEs (Figure 5B). While no specific preference was observed for A, an enrichment of Y-R base pairs was observed for G-containing conserved CDEs.

In summary, CDEs that do not correspond to the initially proposed consensus are conserved across mammals. These conserved CDEs are enriched for high folding probability and show a pronounced preference for U at position 3 of the triloop. Additionally, the identity of the purine base at position 2 in conjunction with the polarity of the closing base pair seems to modulate the repressive strength of CDEs.

Based on evolutionary conservation, a folding probability >5% and a U at position 3 in the triloop, we defined a high-confidence set of 160 Roquin targets with 1–2 CDEs in their 3′UTRs (Supplementary Table S6). To verify the functionality of the newly identified CDEs, we selected three single and one tandem CDE and tested their ability to reduce gene expression in our luciferase reporter system. The individual CDEs of *CSF3* (also *G-CSF*), *CYR61* (also *CCN1*) and *ZC3H12A* (also *Regnase-1* or *MCPIP-1*) and the tandem CDE of *TM2D3*, together with ∼ 40 bp of flanking sequences at each side, were fused to firefly luciferase (Supplementary Figure S13). Furthermore, we introduced point mutations in the closing base pairs of the CDEs to prevent triloop formation and inhibit Roquin binding. As a positive control, the *TNF* CDE and a corresponding mutant were also fused to firefly luciferase. Luciferase activity was reduced to 67–82% in all single CDEs tested, comparable to the effect observed for the single *UCP3* mutants (Figure 5C, compare Figure 3C). The tandem CDE of *TM2D3* reduced luciferase activity more strongly (47%), comparable to the tandem CDE of *UCP3*. All mutants showed significantly higher luciferase activity than the corresponding wt sequences. This shows that new single and tandem CDEs were accurately detected by our bioinformatic prediction.

To verify this finding in an endogenous context, we analyzed changes in mRNA abundance for these four new Roquin targets plus for eight more from the high confidence set, after siRNA-mediated knockdown of Roquin-1 and Roquin-2 (Figure 5D and E; Supplementary Figure S14). We performed this analysis in HEK293 cells and additionally in HUVECs (human umbilical vein endothelial cells), as some of the predicted targets are known to alter the function of endothelial cells. All 12 genes could be verified as Roquin targets in HUVECs and showed a significant increase in mRNA levels after knockdown, which is consistent with increased mRNA stability in the absence of Roquin. This confirms that our bioinformatic prediction is highly accurate in the discovery of new Roquin targets. Notably, only seven of the ten mRNAs detected in HEK293 cells show increased abundance after Roquin knockdown. This shows that not only the expression of Roquin targets is cell-type-specific, but also that their repression by Roquin might be dependent on the cell type.

Recently, stem-loops capped with a U-rich hexaloop were identified by SELEX (systematic evolution of ligands by exponential enrichment) as new high-affinity binding sites for Roquin (37). One such ADE was identified in the *Ox40* 3′UTR, so far the only known example. We searched all human 3′UTRs for additional ADEs to investigate the occurrence of this type of Roquin binding site in other 3′UTRs. The consensus consists of a 6–8 bp long stem capped with a GUUYUA hexa-nucleotide loop (Figure 5F). We chose this stem in analogy to functional CDEs, as the structure of the Roquin-*Ox40* ADE-complex showed no sequence-specific binding within the stem region.

Scanning all human 3′UTRs predicted that 19 genes contain one potential ADE (Supplementary Table S7). Only one of these has a folding probability <5%. About half of the ADEs have an AU-rich stem and contain a purine stack on the 3′ side, comparable to the occurrence of these features in CDEs. No specific polarity of the closing base pair could be detected. Three 3′UTRs were predicted to encode an additional CDE. However, none of these CDEs is conserved, suggesting that ADEs function independently. Six of the ADEs are also conserved in chimpanzees, mice, dogs and cows, underlining their functional importance. To test whether ADE-encoding mRNAs are regulated by Roquin, we analyzed changes in mRNA abundance for four of these after siRNA-mediated knockdown of Roquin-1 and Roquin-2 in HEK293 cells and HUVECs. All four showed a significant increase in mRNA levels after Roquin knockdown in both HEK293 cells and HUVECs (Figure 5G). This confirms that ADEs were accurately detected by our bioinformatic prediction. Importantly, none of the 3′UTRs contains an additional CDE element, which underlines the self-sufficiency of the ADE as a *cis*-regulatory element.

In summary, the definition of a new consensus for CDEs, together with the genome-wide prediction of ADEs, enabled us to predict and verify a variety of new Roquin targets. It is important to note that many of the newly predicted and verified targets are not associated with immune responses, suggesting that new cellular functions of Roquin proteins still need to be investigated.

## DISCUSSION

The three-dimensional structures of RNAs are essential to their physiological function. Thus, structural conservation, equal to sequence conservation, is indicative for functional importance. Bioinformatic prediction of conserved structures in UTRs using a program, Dynalign, that is able to predict conserved structures, independent of prior sequence alignments, allowed us to discover new functional structured elements.

We discovered two CDEs in the 3′UTR of *UCP3*, a family member of five mitochondrial membrane proteins involved in cell metabolism. UCPs are encoded in the cell nucleus and imported into mitochondria after translation of the entire protein in the cytoplasm (53). The first identified uncoupling protein, UCP1, is primarily expressed in brown adipose tissue. Its physiological function is to mediate a regulated, thermogenic proton leak (47). Inducible proton conductance is a feature common to all UCPs. Since proton leakage accounts for a substantial part of the resting metabolic rate, this process represents a potential target for the treatment of metabolic diseases. The physiological role of UCP3 is still under debate (46,54). It is selectively expressed in skeletal muscle, brown fat and diabetic heart. Although it has uncoupling properties, this does not seem to be its primary function. Increased UCP3 levels do not necessarily lead to an increase in uncoupling. Further, the thermoregulation of UCP3^−/−^ mice is not impaired, suggestive of other cellular functions. Several experiments indicate a role of UCP3 in the metabolism of fatty acids. Interestingly, elevated UCP3 levels were observed in obesity-resistant mice and a decrease in UCP3 levels was associated with insulin sensitivity, a condition that precedes diabetes. Thus, modulation of UCP3 levels is an interesting target for the development of novel therapeutics. Fasting, acute exercise and high-fat intake all lead to increased UCP3 levels, which is in line with the induction of *UCP3* caused by PPAR transcription factors (55). Notably, *UCP3* mRNA levels are elevated in fasted mice and are quickly restored after re-feeding, indicating that *UCP3* mRNA is unstable (56). Thus, rapid constitutive decay of *UCP3* mRNA by Roquin’s recruitment of the deadenylase machinery could be essential to enable a rapid response to changing nutritional states.

The *UCP3* CDEs do not correspond to the initially proposed consensus for Roquin-mediated mRNA decay (26). The subsequent detailed mutational analysis showed that Roquin binding is essentially sequence-independent, in accordance with previous structural analyses (33–36). This prompted us to formulate a new consensus for active CDEs. The new consensus enabled us to discover and verify a large number of new Roquin targets. Some of these are in line with the function of Roquin in immune response and inflammation, such as the verified proinflammatory factors CSF3, CYR61 and TNFSF15 (also TL1A). But most suggest a broader cellular function of Roquin that is consistent with the perinatal lethality of Roquin-1 or Roquin-2 knockout mice.

The ZnF-containing Regnase-1 (*ZC3H12A*) was previously identified to detect similar hairpin structures in 3′UTRs (57). Regnase-1 destabilizes its targets by endonucleolytic cleavage and shares an overlapping set of targets with Roquin. Shared targets can either be suppressed cooperatively (58) or through spatiotemporally distinct mechanisms (57). In accordance with the redundant recognition of hairpin structures, we also identified targets previously associated with Regnase-1, such as *TM2D3* and *TFRC* (57,59). In addition, it was shown that Regnase-1 itself is a Roquin target (58). The 3′UTR encodes a functional CDE (60), which, together with the CDEs within the 3′UTRs of Roquin-1 and Roquin-2 (26), allows for extensive auto- and cross-regulation between these three proteins (58,61). Thus, Regnase-1 targets can be regulated either directly by Roquin or indirectly by modulation of Regnase-1 mRNA abundance. In summary, apart from the functions in the immune response, Roquin will also share other cellular functions with Regnase-1, such as the regulation of the iron metabolism.

The *UCP3* 3′UTR contains two closely spaced CDEs, both of which are necessary for efficient Roquin binding and gene repression. Similarly, the *ICOS* 3′UTR contains two closely spaced Roquin binding sites, both necessary for efficient Roquin binding and ICOS repression (32). Repression is additionally supported by a remote CDE that works in conjunction with the tandem binding site. Thus, several Roquin binding sites might act cooperatively in Roquin binding *in vitro* and gene regulation *in vivo*. It is unclear how such cooperativity would be achieved at the molecular level. In solution, Roquin protein is a monomer. However, interaction between Roquin proteins could be induced by RNA binding or by auxiliary factors such as the recently discovered NUFIP2 protein, which enhances *ICOS* repression by Roquin (32).

Similar to the *UCP3* 3′UTR, the *TM2D3* 3′UTR contains two closely spaced CDEs. Both tandem CDEs reduce luciferase activity in our reporter system significantly more than any of the tested single CDEs. Thus, gene suppression by CDEs is somewhat digital in isolation, in that the levels of repression are discrete and relate to the number of CDEs, with two CDEs being more repressive than one CDE. In contrast, the induction of mRNA levels after Roquin knockdown varies greatly between different target genes and also between cell types. The largest induction is observed for the cytokine CSF3. However, *CSF3* CDE alone has no greater influence on luciferase expression than other individual CDEs.

Interestingly, the *CSF3* CDE was characterized as a repressive element even before Roquin was discovered. Mutational analysis of the *CSF3* CDE showed the same preference for a YAU triloop and no Roquin binding after prevention of triloop formation (62). Notably, the closing base pair in the *CSF3* CDE is A-U, as in both *UCP3* CDEs. Thus, although YRN triloops and any closing base pair are tolerated in active CDEs, some sequence combinations seem to be better recognized by Roquin. The reason for these preferences is still unclear. Up to now, all high-resolution structures of Roquin RNA complexes use a UGU triloop with a C-G closing base pair (33–36). Here the middle G of the triloop is stacked between the G of the closing base pair and arginine 219. An A at this position could allow for different interactions within the CDE or with the ROQ domain and thus ultimately support complex formation. Alternatively, specific sequence combinations could support preformation of the CDE to facilitate conformational selection by Roquin. In this context, it was shown that incorrect preformation inhibits Roquin binding to a mutant of the *TNF* CDE (50).

In addition to CDEs, ADEs have emerged as functionally important Roquin binding sites. Interestingly, two of the verified targets, MAP3K5 (also ASK1) and Itch, were already known targets of Roquin. The stability of the MAP3K5 protein is regulated through ubiquitination by Roquin-2 (63). Our study suggests an additional layer of regulation by mRNA destabilization. Itch has recently been identified as a downstream target of Roquin in T cells (64). The 3′ half of the *Itch* 3′UTR was sufficient to confer Roquin-dependent repression. This is where the predicted *Itch* ADE is located, emphasizing the accurate prediction of functional ADEs.

Further determinants will modulate the response of individual target genes to changes in Roquin protein levels. One of these is the folding probability of the CDE/ADE within its 3′UTR, with a high folding probability facilitating conformational selection by Roquin. In addition, auxiliary proteins such as the recently described NUFIP2 (32) or auxiliary sequences such as other stem loop elements with even larger U-rich loops recognized by the ROQ domain (27,32,64), or AU-rich sequences recognized by the ZnF (27), would support Roquin binding. Other determinants that will modulate regulation by Roquin are competing factors that also bind to CDEs. The binding of Regnase-1 could destabilize shared target mRNAs in the absence of Roquin, so that they remain partially suppressed (61). Conversely, interactions with antagonists such as ARID5A (65) and BAG3 (66) would further enhance the stability of the target mRNA. In addition, backup systems independent of the CDE could also ensure continuous destabilization of the mRNA. These could be AU-rich elements, as for example in the *TNF* 3′UTR (67), or miRNA binding sites. In this context, it has been shown that Roquin cooperates with miR-146a in *ICOS* mRNA repression (68). Notably, the *UCP3* 3′UTR also contains miRNA binding sites directly upstream of its tandem CDE. Thus, Roquin could also support the suppression of UCP3 by these miRNAs.

As increasingly complex patterns of post-transcriptional gene regulation are discovered, it will become more and more important to integrate different genome-wide methods with a detailed mutational analysis in order to clarify the exact binding preferences of individual RNA binding proteins. In this context, we have successfully implemented genome-wide bioinformatic predictions of conserved structured elements for the discovery of new Roquin targets. This discovery led to a revised consensus for functional CDEs and uncovered new cellular functions of Roquin. Thus, *in silico* predictions of RNA structures are both highly complementary and informative for *in vivo* experiments. Future work should focus on the importance of auxiliary Roquin binding sites and the effect of competing proteins to provide accurate predictions of target gene responses to fluctuating Roquin levels and thus advance our understanding of the role of Roquin in health and disease.

## Supplementary Material

Supplementary Data
