# Supplementary material for: Identification of new high affinity targets for Roquin based on structural conservation
Source: Nucleic Acids Res. 2018 Oct 8;46(22):12109–25. doi: 10.1093/nar/gky908 (PMC6294493; doi:10.1093/nar/gky908)
Supplement: Supplementary Data [file gky908_supplemental_files.zip › Supplement revised.pdf]

Identification of new high affinity targets for Roquin based on structural conservation

Johannes Braun[1], Sandra Fischer[1], Zhenjiang Zech Xu[2], Hongying Sun[2], Dalia H. Ghoneim[2], Anna Theresa Gimbel[1], Uwe Plessmann[3], Henning Urlaub[3,4], David H. Mathews[2] and Julia E. Weigand[1,*]

[1] Department of Biology, Technische Universität Darmstadt, Darmstadt, 64287, Germany
[2] Department of Biochemistry and Biophysics and Center for RNA Biology, University of Rochester Medical Center, Rochester, New York, 14642, USA
[3] Biophysical Mass Spectrometry Group, Max Planck Institute for Biophysical Chemistry, Göttingen, 37077,
[4] Bioanalytics, Institute for Clinical Chemistry, University Medical Center, 37073 Göttingen, Germany

* To whom correspondence should be addressed. Tel: +49 6151 16 22005; Fax: +49 6151 16 22003; Email: weigand@bio.tu-darmstadt.de

Supplementary Material

Supplementary Methods

Supplementary Figure 1. Structurally conserved regions predicted by Dynalign influence gene expression.

Supplementary Figure 2. The structurally conserved region of the *UCP3* 3'UTR encodes a functional miRNA binding site.

Supplementary Figure 3. Gene regulation is independent of the high AU content of the *UCP3* wt element.

Supplementary Figure 4. The *UCP3* wt element reduces mRNA abundance.

Supplementary Figure 5. Identification of proteins associated with the *UCP3* wt element.

Supplementary Figure 6. Roquin mediates repression by the *UCP3* wt element.

Supplementary Figure 7. Roquin knockdown reduces *UCP3* mRNA levels in C2C12 cells.

Supplementary Figure 8. Secondary structure prediction of *UCP3* RNAs used for Roquin binding experiments.

Supplementary Figure 9. The ROQ domain is essential for binding of Roquin-1 to the *UCP3* wt element.

Supplementary Figure 10. Mutational analysis of the *UCP3* tandem CDE.

Supplementary Figure 11. Conservation of the CDEs in *UCP3* across mammals.

Supplementary Figure 12. Probability of folding and composition of the stem of conserved CDEs.

Supplementary Figure 13. Overview of conserved CDEs in the 3'UTRs of new Roquin targets.

Supplementary Figure 14. siRNA efficiency in HEK293 cells and HUVECs.

Supplementary Table 1. *UCP3* tandem CDE sequences and corresponding RLUs.

Supplementary Table 2. Oligonucleotide sequences used for reverse transcription and qPCR analysis.

Supplementary Table 3. Conserved 3'UTR structures predicted by Dynalign and tested for functionality *in vivo*.

Supplementary Table 4. Cycle thresholds of miRNAs predicted to bind to the *UCP3* element.

Supplementary Table 5. Proteins enriched by RNA affinity purification.

Supplementary References

Supplementary Methods

Reverse transcription and qPCR

Sequence-specific stem-loop (SL) primers were used to enable target specific reverse transcription of miRNAs. For miRNA quantification, 100 ng of DNase-treated RNA were transcribed into cDNA. For normalization in qPCR, the small nucleolar RNA (snoRNA) U48 was co-transcribed to cDNA. The reaction mixture/sample contained 1x First-strand buffer (Invitrogen), 50 nM of each SL primer (Sigma Aldrich), 10 µM DTT, 1 µM dNTPs (0.25 µM each), 5 U/µl Superscript II (Invitrogen) in a 20 µl total reaction volume. The program for reverse transcription contained the following steps: 16°C for 30 min; 60 cycles: 30°C for 30 s, 42°C for 30 s, 50°C for 1min; 70°C for 15 min. The qPCR program consisted of the following steps: 50°C for 2 min; 95°C for 10 min; 40 cycles: 95°C for 15 s, 60°C for 1 min. The reaction mixture contained: 1x TaqMan Universal PCR Master Mix (Applied Biosystems), 0.5 µM Universal primer and miRNA-specific qPCR primer, 1 µl Universal Probe #21 (Roche), 10 ng cDNA in a 20 µl reaction volume.

For mRNA analysis, total RNA was reverse transcribed to cDNA using MuLV reverse transcriptase (Thermo Fisher Scientific). The reaction mixture contained 1x PCR Buffer II (Thermo Fisher Scientific), 5 mM $MgCl_2$, 0.5 µM random hexamers (Sigma Aldrich), 1 mM dNTPs (0.25 mM each), 2,5 U/µl MuLV (Thermo Fisher Scientific) and 2U/µl Ribolock (Molox) in a 40 µl total reaction volume. The program for reverse transcription contained the following steps: 20°C for 10 min; 42°C for 15min and 99°C for 5 min. The qPCR program consisted of the following steps: 95°C for 20 s; 40 cycles: 95°C for 3 s, 60°C for 20 s; 60°C for 1 min. qPCR reactions were set up in 96 well plates and contained 1x Fast SYBR Green Master Mix (Thermo Fisher Scientific), 25 ng cDNA, 0.5 µM gene-specific forward and reverse oligonucleotides in a 20 µl reaction. qPCR was performed using the Applied Biosystems StepOne Plus Real-Time PCR System (Applied Biosystems).

In-line probing

For in-line probing, RNA was dephosphorylated and 5' ^{32}P-labeled as described (1). After PAGE purification, 40 kcpm of the 5' ^{32}P-labeled RNA were incubated for 60 h at 22°C in in-line reaction buffer (10 mM Tris-HCl pH 8.3, 10 mM $MgCl_2$, 100 mM KCl). To generate a size marker, the 5' ^{32}P-labeled RNA was subjected to alkaline hydrolysis by incubation for 3 min at 96°C in 50 mM Na_2CO_3 pH 9.0. To identify guanines, the 5' ^{32}P-labeled RNA was incubated for 3 min at 55°C with 20 U RNase T1 (Ambion). After in-line reaction, alkaline hydrolysis or RNase T1 treatment, reactions were ethanol precipitated and the pellet was dissolved in 5 M urea. Reaction products were separated by denaturing PAGE. After drying, gels were analyzed using phosphoimaging (GE Healthcare).

Electromobility shift assays (EMSAs)

For the binding reaction, a master mix containing tRNAs, ^{32}P-labeled CDE RNA and reaction buffer was prepared and then mixed with dilutions of the recombinant proteins to achieve the indicated protein concentrations. The binding was performed for 10 min at room temperature in a 20 µl reaction volume in the presence of 2.5 µg/µl yeast tRNA (Sigma Aldrich), 500 pmol ^{32}P-labeled RNA, 20 mM

HEPES pH 7.4, 50 mM NaCl, 1 mM $MgCl_2$, 1 mM DTT and 1 µg/µl BSA. After addition of 4 µl 30% (v/v) glycerol as loading buffer, the RNP complexes were resolved by PAGE (6% polyacrylamide, 5% glycerol, and 0.5x TBE) at 120 V for 40 min at room temperature. After drying, gels were analyzed using phosphoimaging (GE Healthcare). The bound fraction was normalized to the total signal for each lane and plotted against the protein concentration. A nonlinear regression curve was fitted to the data points with the equation $Y=B_{max}*X^h/(K_d^h + X^h)$ in which B_{max} is the maximum specific binding, and h is the Hill coefficient.

Computational methods

a) Search for conserved structures using Dynalign:

The UTR sequences of human chromosomal DNA were acquired from the genome build hg18, downloaded from UCSC genome browser (http://hgdownload.soe.ucsc.edu/downloads.html#human). These UTRs were fragmented into overlapping windows of 100 nt with a step size of 50 nt. The corresponding mouse genomic sequences of those 100 nt windows were then identified using the human-mouse pairwise genomic alignment obtained from UCSC genome browser (http://hgdownload.soe.ucsc.edu/goldenPath/hg18/vsMm9/).
Each window pair of human and mouse sequences were input to Dynalign for RNA structure prediction (2). Then Dynalign/SVMz was used to classify the results by their probability of being a conserved structure (3). First, a measurement of the significance of the calculated free energy changes, z-score, was calculated as compared to shuffled sequences (Uzilov et al., 2006). The Z-score, the number of standard deviations from the mean, is calculated as:

$$z = \frac{m - \mu}{\sigma}$$

where m is the predicted minimum free energy change of the input, µ and σ is the mean and the standard deviation of free energy changes of the shuffled sequences. Second, the Structure Conservation Index (SCI) is calculated (4):

$$SCI = \frac{\Delta G^\circ_{dynalign}}{\Delta G^\circ_{seq1} + \Delta G^\circ_{seq2}}$$

where $\Delta G^\circ_{dynalign}$ is the Dynalign predicted total minimum free energy change of the two sequences and ΔG°_{seq1} and ΔG°_{seq2} are the predicted minimum free energy change for each sequence alone (5). SCI is an indicator of structural conservation. The larger the SCI, the more conserved the secondary structures are. Using the stability z-score and SCI, with the nucleotide composition of the two input sequences, i.e. the frequencies of A, U, G and C, a support vector machine, called SVMz, was trained to classify with the probability that a window is a conserved structure. The windows of sequence pairs were ranked by their probability of being a conserved structure and the windows with probability greater than 0.9 were further analyzed.

b) Prediction of human CDEs and their folding probability

The whole human genome sequence (hg19) was downloaded from UCSC genome browser and the sequence was processed to 3'UTRs using bam2x (https://github.com/nimezhu/bam2x). CDEs are extended by 200 nucleotides both upstream and downstream. RNAmotif, which takes an RNA descriptor, an RNA sequence file and outputs the positions where the RNA motif is found (6), was run to find genes that contain the desired motif. The motif is an RNA stem-loop which has a stem with a length of 6-8 base pairs and a triloop with the sequence 5' YRN 3'. Y stands for C or U; R stands for A or G; and N could be any of the four nucleotides (ACGU). Then rm2ct from the RNAmotif program was used to convert an output file to a ct file, which indicates the sequence and base pairing. The efn2 program from RNAstructure (7), which takes a ct file and calculates the folding free energy change, was run and the equilibrium constant for the interior part of the desired motif is calculated using the following equation:

$$K_{interior} = \mathrm{e}^{-\frac{\Delta G_{37}^{0}}{RT}} \quad (1)$$

where R is the gas constant and T is the absolute temperature in K. The probability of a certain motif occurring in a given RNA sequence is as follows:

$$P_{motif} = K_{interior} * Q_{exterior}/Q \quad (2)$$

where Q exterior is the partition function over configurations outside the motif and Q is the total partition function (8). In order to calculate the exterior probability, a C++ program called ProbStemloop, was written using RNAstructure. ProbStemloop takes three input parameters: the sequence file, the starting position of a stem-loop, the length for the stem-loop and outputs the exterior probability for the motif. The sequences processed by ProbStemloop were truncated to 400 nt upstream and 400 nt downstream. Prior work demonstrated this much sequence is a sufficient representation of the local folding in mRNA (9). Finally, the probability of the motif is calculated with equation 2. All the code, written in python and C++, is available at https://github.com/hongyingsun1101/Motif-Probability.

Similarly, stem-loops containing a hexaloop were searched across the human genome and the probability of this hexaloop containing stem-loops was also calculated. The motif is an RNA stem-loop which has a stem with a length of 6-8 base pairs and a hexaloop with the sequence 5' GUUYUA 3'.

c) Evolutionary conservation of putative CDEs

The RefSeq genomic coordinates and sequences for mRNA 3' UTRs containing predicted CDEs were obtained from UCSC Table browser (10) using the hg19/GRCh37 reference genome. Because of occasional inconsistencies in UCSC and NCBI RefSeq genomic coordinates for 3'UTRs, UCSC nucleotide sequences were scanned to identify the respective CDE and genomic coordinates of the CDEs were adjusted as needed.

Multiz 46-way (11) multiple species alignments across each chromosome were obtained from UCSC golden path database (updated 30-Oct-2009). For each instance of the CDE motif, we determined conservation using multiple sequence alignments that fully span the length of the motif. We checked for exact sequence conservation of each hg19 CDE across *Mus musculus* (mm9, July 2007), *Pan troglodytes* (panTro2, Mar 2006), *Bos taurus* (bosTau4, Aug 2006), and *Canis familiaris* (canFam2, May 2005). In the cases where there are gaps at the 5' or 3' end of a CDE in any of the species, the nucleotides 5' or 3' to the gap, respectively, were used to fill in the gap. In addition, we checked for conservation across the five species allowing tolerated changes. Tolerated changes were defined as any of the following: *(a)* changes in the triloop region that still fit the definition of the motif (5' stem - Y R N - stem 3'), *(b)* changes to the length of the stem such that only 6 base pairs are required to be conserved, and changes to the stem that can still form canonical base pairs. These could be *(c)* compensating base pair changes, or *(d)* G◦U wobble base pairs.

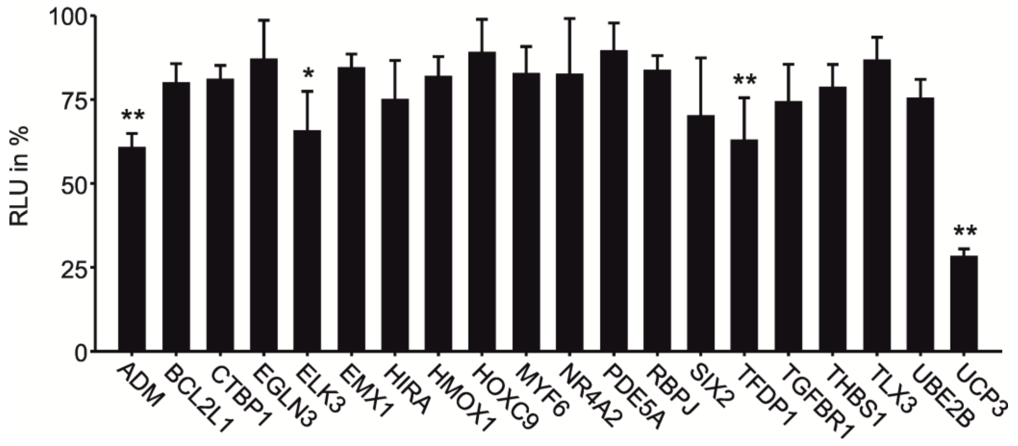

Supplementary Figure 1. Structurally conserved regions predicted by Dynalign influence gene expression. Luciferase activity of various 3’UTR fusion constructs. 100 nt long 3’UTR sequences predicted to encode structurally conserved regions, were fused to firefly luciferase. Firefly luciferase activity was normalized to *Renilla* luciferase as internal transfection control. Values are normalized to an empty vector control. n = 3. (**) P-value <0.01. (*) P-value <0.05.

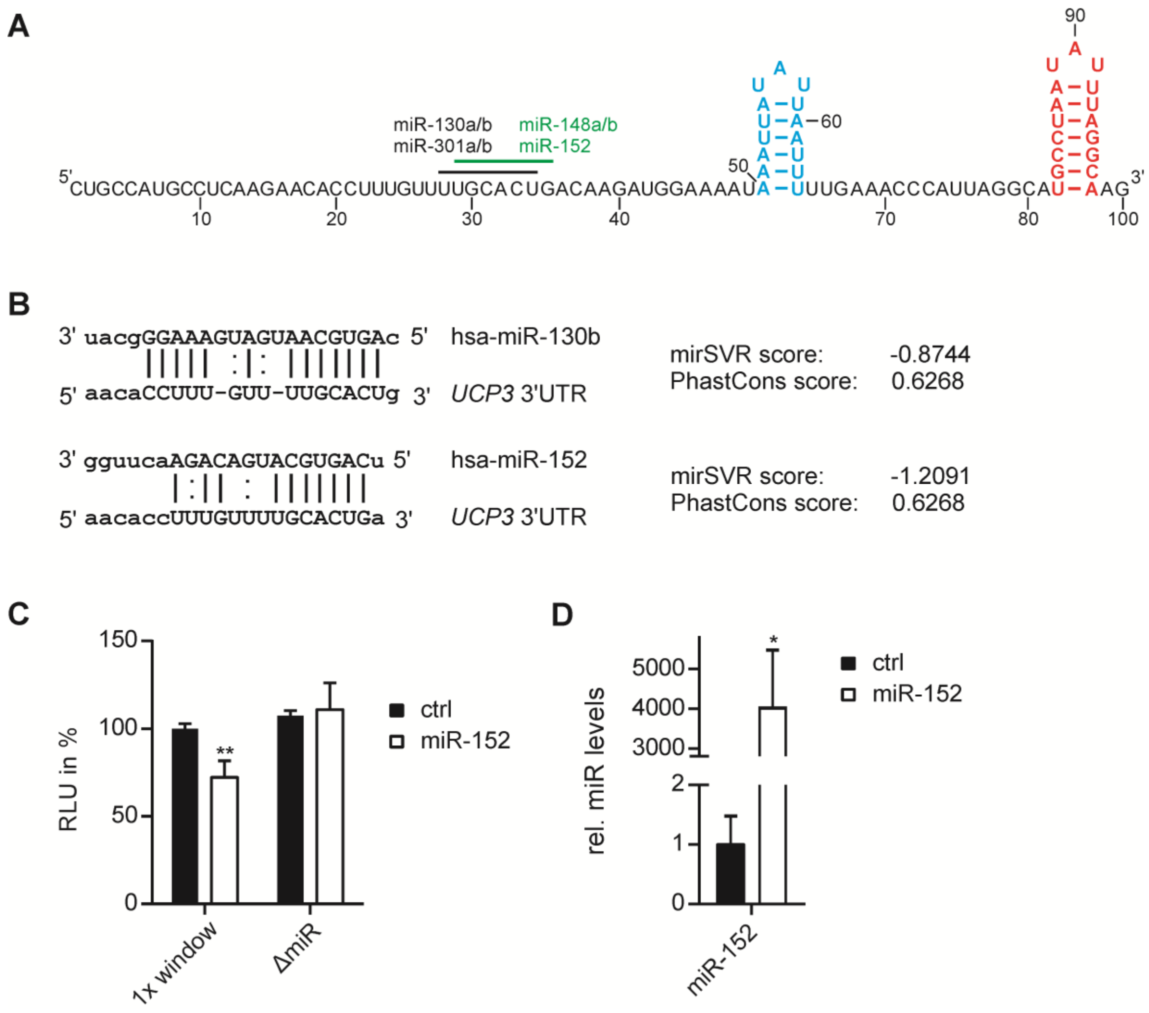

Supplementary Figure 2. The structurally conserved region of the *UCP3* 3'UTR encodes a functional miRNA binding site. (**A**) Location of the miRNA binding site within the Dynalign window. The window encodes two overlapping sites indicated by black and green lines, respectively. (**B**) Predicted miRNA target sites by miRanda (http://www.microrna.org/). Shown are the taget site alignement as well as miRanda-SVR calculated miR-SVR scores and target site conservation by PhastCons (12). (**C**) Luciferase activity of the *UCP3* Dynalign element (1x window) and a construct in which both miRNA binding sites are deleted (ΔmiR) with and without overexpression of miR-152. Firefly luciferase activity was normalized to *Renilla* luciferase as internal transfection control. Values are normalized to an empty vector control. n = 3. (**D**) RT-qPCR quantification of miR-152 levels after overexpression. Values are normalized to the snoRNA U48 as internal control. (**) P-value <0.01. (*) P-value <0.05.

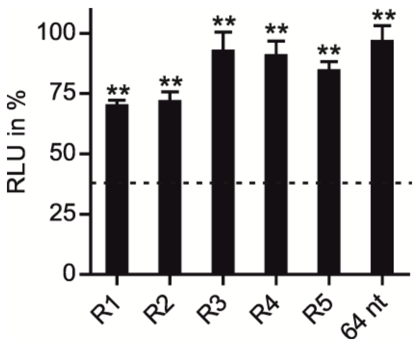

Supplementary Figure 3. Gene regulation is independent of the high AU content of the *UCP3* wt element. Luciferase activity of randomized sequences with the same nucleotide composition (R1-R5) as the *UCP3* wt element and one randomized sequence with uniform nucleotide content (64 nt). Firefly luciferase activity was normalized to *Renilla* luciferase as internal transfection control. Values are normalized to an empty vector control. n = 3. (**) P-value <0.01.

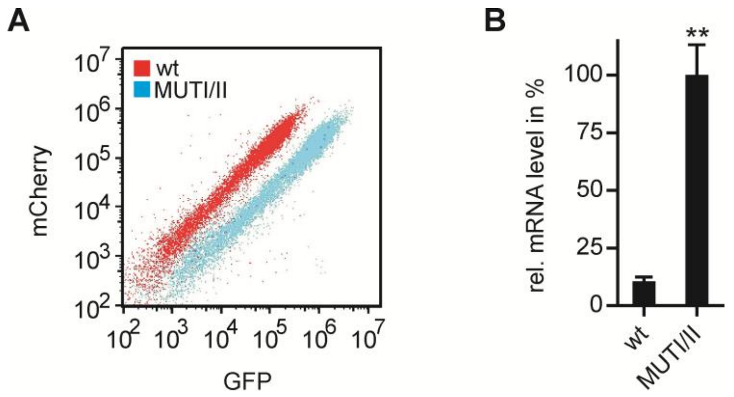

Supplementary Figure 4. The *UCP3* wt element reduces mRNA abundance. (**A**) GFP and mCherry fluorescence of *UCP3* wt and double mutant (MUTI/II). GFP-UCP3-fusion constructs were stably integrated into the genome of HeLa cells. GFP and mCherry fluorescence were measured by flow cytometry. n = 3. (**B**) RT-qPCR quantification of *GFP* mRNAs containing the *UCP3* wt element (wt) or double mutant (MUTI/II). Total RNA from HeLa cells stably expressing one of the two constructs was isolated and *GFP* mRNA levels quantified by RT-qPCR. *GFP* values are normalized to the housekeeping gene *RPLP0*. n = 3. (**) P-value <0.01.

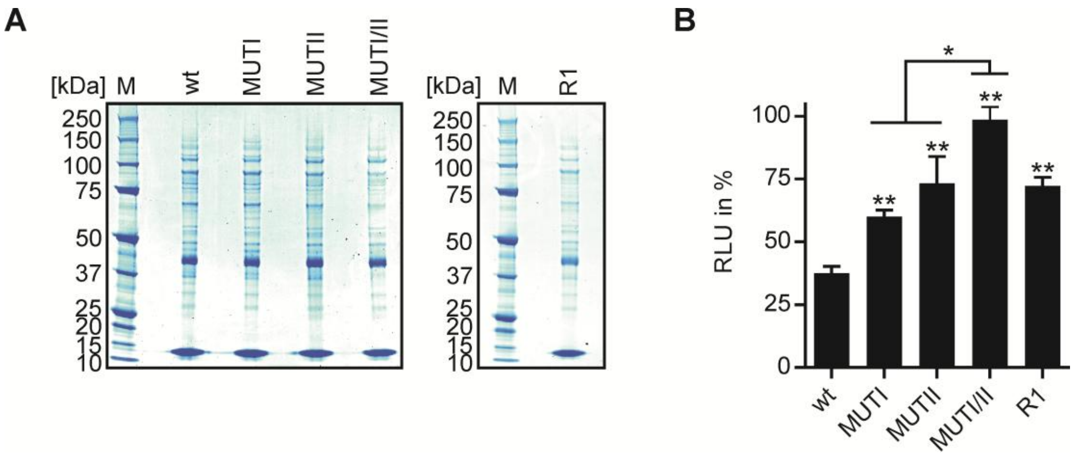

Supplementary Figure 5. Identification of proteins associated with the *UCP3* wt element. (**A**) Analysis of protein binding to the *UCP3* RNAs shown in (**Figure 2F**) and a randomized control with the same nucleotide composition (R1). For RNA affinity purification HEK293 whole cell lysates were incubated with the different *UCP3* RNAs. Associated proteins were visualized by SDS-PAGE. M = protein size marker. (**B**) Luciferase activity of *UCP3* constructs (shown in **Figure 2F**) and a randomized control with the same nucleotide composition (R1) used for RNA affinity purification. Firefly luciferase activity was normalized to *Renilla* luciferase as internal transfection control. Values are normalized to an empty vector control. n = 3. (**) P-value <0.01. (*) P-value <0.05.

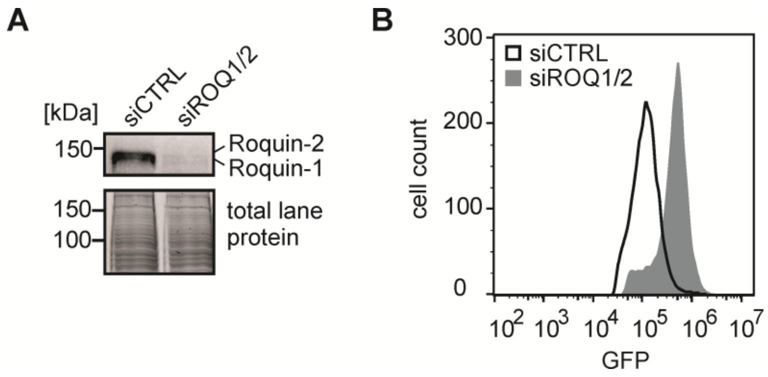

Supplementary Figure 6. Roquin mediates repression by the *UCP3* wt element. (**A**) Western Blot of Roquin-1 and Roquin-2 after siRNA-mediated knockdown in HeLa cells stably expressing a *GFP* mRNA containing the *UCP3* wt element. Anti-Roquin was used to verify the respective knockdown. Total lane protein is shown as loading control. n = 3. (**B**) GFP fluorescence of *UCP3* wt with and without siRNA-mediated knockdown of Roquin-1 and Roquin-2. GFP fluorescence was measured by flow cytometry. n = 3.

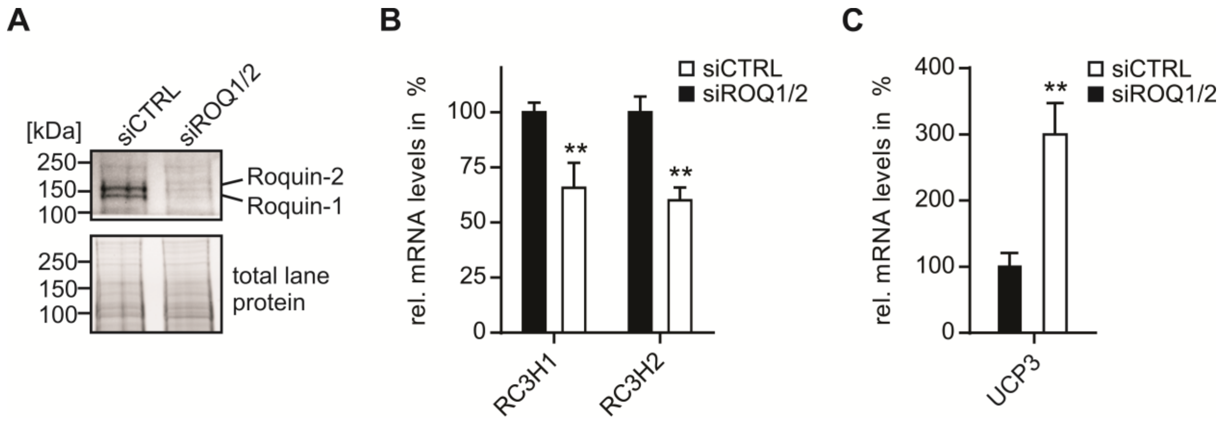

Supplementary Figure 7. Roquin knockdown reduces *UCP3* mRNA levels in C2C12 cells. (**A**) Western Blot of Roquin-1 and Roquin-2 after siRNA-mediated knockdown in C2C12 cells. Anti-Roquin was used to verify the respective knockdown. Total lane protein is shown as loading control. (**B and C**) RT-qPCR quantification of (**B**) *RC3H1* and *RC3H2* and (**C**) *UCP3* mRNA levels after siRNA-mediated knockdown of Roquin-1 and Roquin-2 in C2C12 cells. Values are normalized to the housekeeping gene *GAPDH*. n = 3. (**) P-value < 0.01.

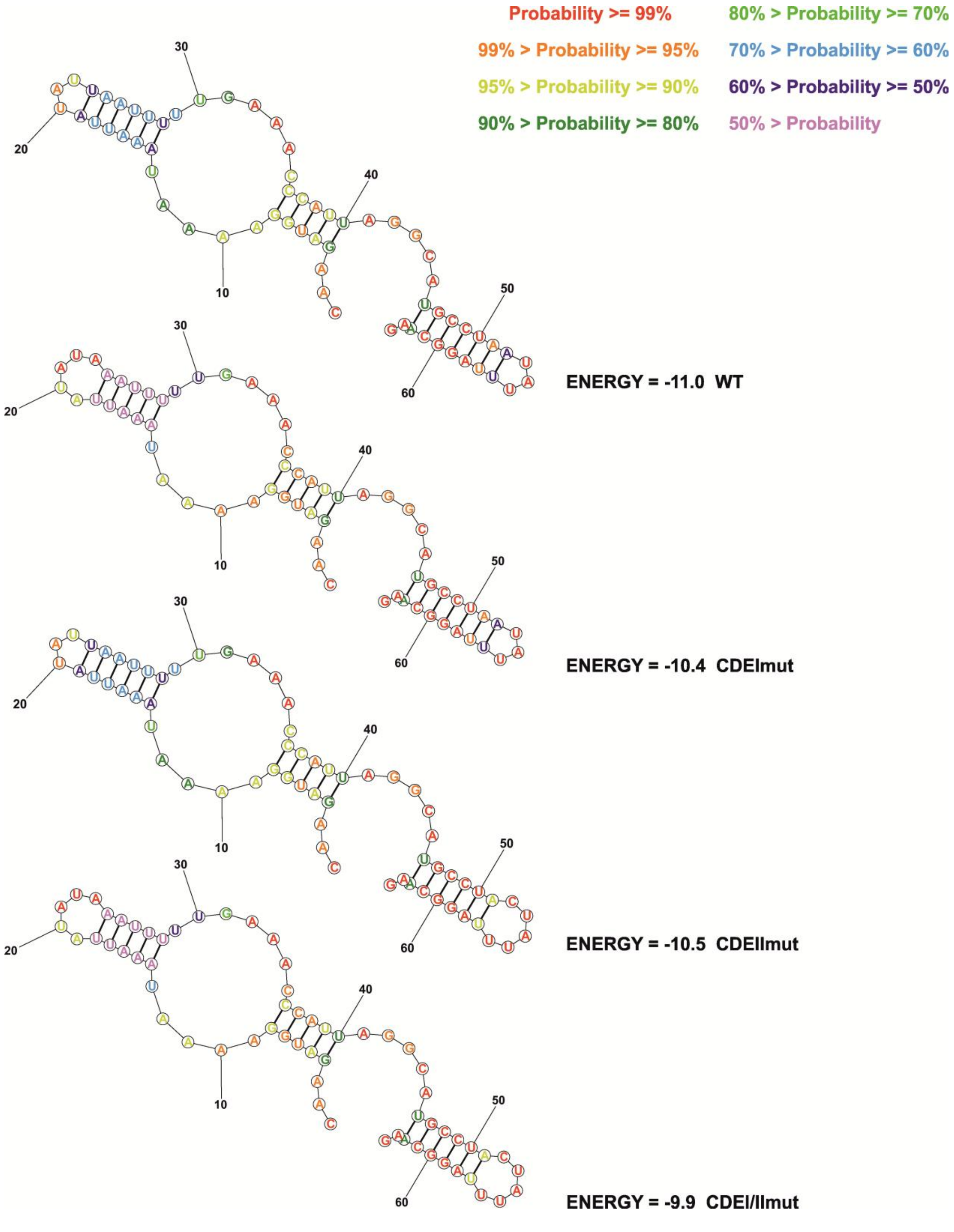

Supplementary Figure 8. Secondary structure prediction of *UCP3* RNAs used for Roquin binding experiments. Secondary structure of *UCP3* constructs used for EMSAs with recombinant Roquin-1 protein predicted by RNAstructure v6.0.1 (https://rna.urmc.rochester.edu/RNAstructureWeb).

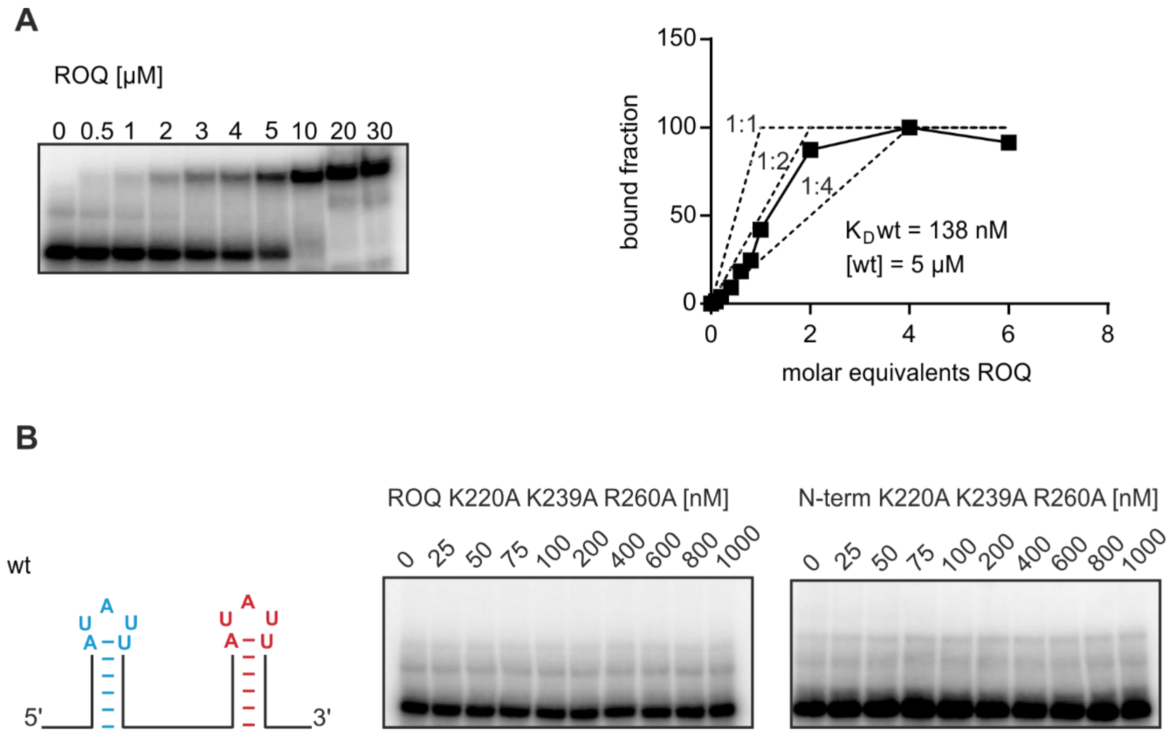

Supplementary Figure 9. The core ROQ domain is essential for binding of Roquin-1 to the *UCP3* wt element. (**A**) Left: Increasing amounts of Roquin-1 ROQ domain were incubated with 5 μM *UCP3* wt RNA containing a trace of radioactively labeled RNA. Right: Comparison of the observed binding saturation with theoretical saturation curves for a 1:1; 1:2 and 1:4 binding stoichiometry. n = 2. (**B**) Radiolabeled *UCP3* wt RNA was incubated with increasing amounts of a triple mutant of Roquin-1 ROQ domain or N-terminus. n = 2.

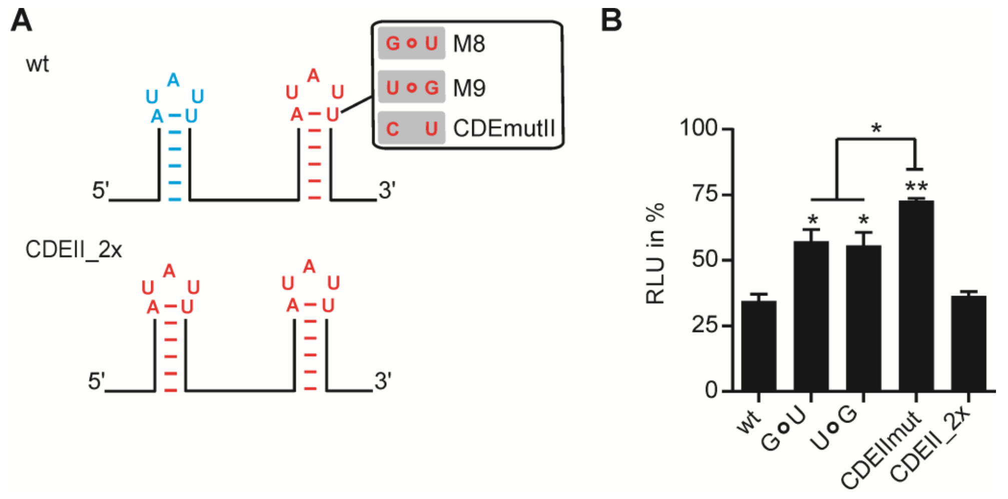

Supplementary Figure 10. Mutational analysis of the *UCP3* tandem CDE. (**A**) Overview of *UCP3* mutants. (**B**) Luciferase activity of *UCP3* mutants. Firefly luciferase activity was normalized to *Renilla* luciferase as internal transfection control. Values are normalized to an empty vector control, without *UCP3* 3'UTR sequences. n = 3. (**) P-value <0.01. (*) P-value <0.05.

```
Human              CAAGAUGGAAA-AUAAAUUAUAUUAAUUUUUGAAACCCAUUAGGC---AUGCCUAAUAUUUAGGCAAG
Chimp              CAAGAUGGAAA-AUAAAUUAUAUUAAUUUUUGAAACCCAUUAGGC---AUGCCUAAUAUUUAGGCAAG
Gorilla            CAAGAUGGAAA-AUAAAUUAUAUUAAUUUUUGAAACCCAUUAGGC---AUGCCUAAUAUUUAGGCAAG
Orangutan          CAAGAUGGAAA-AUAAAUUAUAUUAAUUUUUGAAACCCAUUAGAC---AUGCCUAAUAUUUAGGCAAG
Rhesus             CAAGAUGGAAA-AUAAAUUAUAUUAAUUUUUGAAACCCAUUAGGC---AUGCCUAAUAUUUAGGCAAG
Squirrel           CUUAAUGGAAA-AUAAAUUAUAUUAAUUUU-GAAACCCAUUAGGUUGGAUGCCUAAUAUUUAGGCAAG
Mouse              CCUGAUGGGAA-AUAAAUUAUAUUAAUUUUUAAACCCCUUCCGGUUGGAUGCCUAACAUUUAGGCAAG
Rat                CCAGAUGGGAA-AUAAAUUAUAUUAAUUUUUAAAACCUUUCCGGUUGGAUGCCUAACAUUUAGGCAAG
Guinea pig         CAUGAUGGGAA-AUAAAUUAUAUUAAUUUGUGAAAUGCAUUGGGAUGGGUGCCUAACAUUUAGGCAAG
Chinchilla         CAUGAUGGAAA-AUAAAUUAUAUUAAUUUGUGAAACCCAUUGGGUUGGGUGCCUAACAUUUAGGCAAG
Rabbit             CAAGAUGGAGA-AUAAAUUAUAUUAAUUUUUGAAACUCAUUAGAUUGGAUGCCUAACAUUUAGGCAGA
Pig                CAAGAUGCUCA-AUAAAUUAUAUUAAUUUGUGAAACCCACUAGGAUGGAUGCCUAACAUUUAGGCAAA
Alpaca             CAAGAUGGAAA-AUAAAUUAUAUUAAUUUGUGCAACCUGUUAGGUUGGCCGCCUAACAUUUAGGCAAG
Camel              CAAGAUGGAAA-AUAAAUUAUAUUAAUUUGUGCAACCUGUUAGGUUGGCCGCCUAACAUUUAGGCAAG
Dolphin            CAACAUGGAAA-AUAAAUUACAUUAAUUUGUGAAACCCAUCAGGUUGGAUGCCUAACAUUUAGGCAAG
Cow                CAAGGUGGAAA-AUAAAUUAUAUUAAUUUGUGAAAUCCAUCAGGUUGGAUGCCUAACAUUUAGGCAAG
Sheep              CAAGGUGGAAA-AUAAAUUAUAUUAAUUUGUGAAAUCCAUCAGGUUGGAUGCCUAACAUUUAGGCAAG
Goat               CAAGGUGGAAA-AUAAAUUAUAUUAAUUUGUGAAAUCCAUCAGGUUGGAUGCCUAACAUUUAGGCAAG
Horse              CAAGAUGGAAA-AUAAAUUACAUUAAUUUUUGAAGCCCAUAGGGUUGGAUGCCUAACAUUUAGGCAAG
Rhinoceros         CAGGAUGGAAA-AUAAAUUAUAUUAAUUUUUGAAACCCAUUAGAUUGGAUGCCUAACAUUUAGGCAAG
Cat                CAAGAUGGCAA-AUAAAUUAUAUUAAUUUGGGGGACCCAUUAAGUUGGAUGCCUAAUAUUUAGGCAAG
Dog                CAAGAUGGAAA-AUAAAUUAUAUUAAUUUUGGGGACCUAUUAAGUUGGAUGCCUAACAUUUAGGCAAG
Panda              CAAGAUGGAAA-UUAAAUUAUAUUAAUUUGGGGGACCCAUGAUGUUGGAUGCCUAACAUUUAGGCAAG
Walrus             CAAGAUGGAAA-UUAAAUUAUAUUAAUUUGGGGGACCCAU-AUGUUGGAUGCCUAACAUUUAGGCAAG
Microbat           UAAGAUAGCAA-AUAAAUUAUAUUAAUUUUUGAAACCCAUUAAGUUGGAUGCCUAACAUUUAGGCAAG
Shrew              CAAGAAGGAAA-AUAAAUUAUGUUAAUUUUUGUAACCCACUGGGUUGGAUGCCUAACAUUUAGGCACA
Star-nosed mole    CAAGAUGGAAA-AUAAAUUAUACUAAUUUUUGUAACCUAUCAGGAUGGAUGCCUAACAUUUAGGCAAG
Elephant           CAAGAUGGAAA-AUAAAUUAUAUUAAUUUUUGAAACUCAUUAGGUGGAAUGCCUAACAUUUAGGCAAG
Manatee            CAAGAUGGAAA-AUAAAUUAUAUUAAUUUUUGAAACACAUUAGGUUGGAUGCCUAACAUUUAGGCAAG
Opossum            CAAGAUAAAGUCAUAAAUUAUAUUAAUUUUU-AAACAUAUCAAGAUUGCUGCCUAAUAUUUAGGCAAG
Tasmanian devil    CAAGACAAAAUCAUAAAUUAUAUUAAUUUCUGAAAUAUACCAAGGUUGAUGCCUAAUAUUUAGGCAAG
Platypus           CAAGAUGGAAAUGUAAAUUAUAUUAAUUUGGGAAACACAAUAAGGCU-ACGCCUAACGUUUAGGUCAC
```

Supplementary Figure 11. Conservation of the CDEs in *UCP3* across mammals. CDEI and CDEII are highlighted in blue and red, respectively. Mutations within CDEs relative to the human sequence are indicated in black/bold.

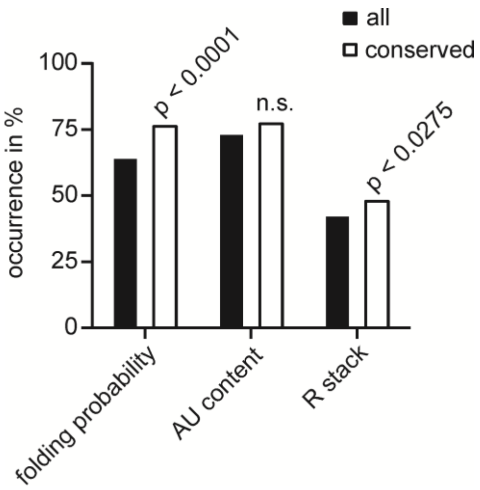

Supplementary Figure 12. Probability of folding and composition of the stem of conserved CDEs. Comparison of folding probability (> 5%), AU content (> 50%) and occurrences of purine (R) stacks at the 3' side of the stem (at least three consecutive purines) between conserved and non-conserved CDEs. p = P-value (Pearson's chi-square test). n.s. = not significant.

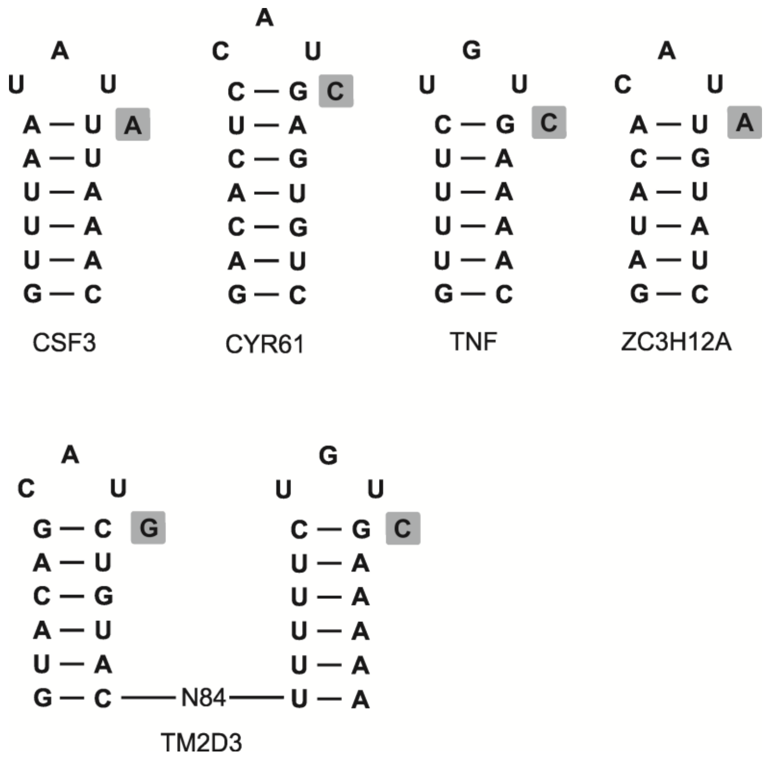

Supplementary Figure 13. Overview of conserved CDEs in the 3'UTRs of new Roquin targets. Inactivating mutations preventing triloop formation are shown next to the respective CDE.

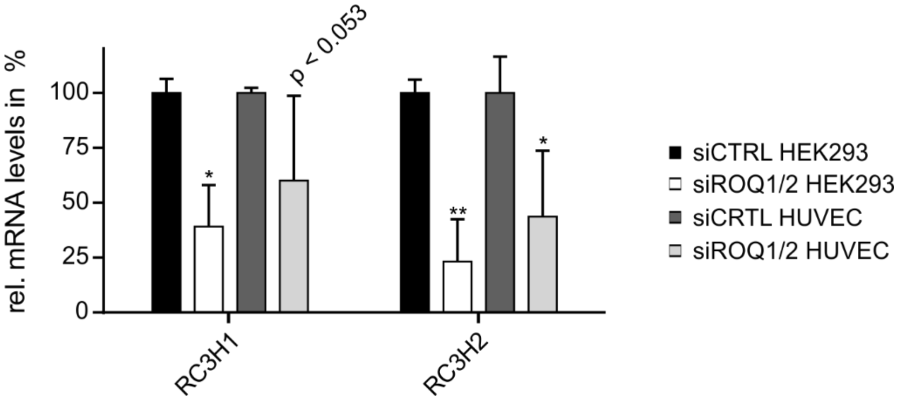

Supplementary Figure 14. siRNA efficiency in HEK293 cells and HUVECs. RT-qPCR quantification of *RC3H1* and *RC3H2* mRNA levels after siRNA-mediated knockdown of Roquin-1 and Roquin-2 in HEK293 cells and HUVECs. Values are normalized to the housekeeping gene *RPLP0*. n = 4. (**) P-value <0.01. (*) P-value <0.05.

Supplementary Table 1. *UCP3* tandem CDE sequences and corresponding RLUs (related to **Figures 1- 4**).

Construct	Sequence 5' → 3'	RLU*
window	CTGCCATGCCTCAAGAACACCTTTGTTTTGCACTGACAAGATGGAAAATA<br>AATTATATTAATTTTTGAAACCCATTAGGCATGCCTAATATTTAGGCAAG	33.2
miRmut	CTGCCATGCCTCAAGAACACCTTTGTTT**--------**CAAGATGGAAAATA<br>AATTATATTAATTTTTGAAACCCATTAGGCATGCCTAATATTTAGGCAAG	36.5
random ctrl	**GAATATAGTATTCATTAGTACATCGCATACTTATCAATATTCTGAGGCAA**<br>**GATAGAGTACTGATCAGAATCTACTTCAATTTCGCGTTACGCCAAATCAG**	77.4
sh1	**---------------------**TTTGTTTTGCACTGACAAGATGGAAAATA<br>AATTATATTAATTTTTGAAACCCATTAGGCATGCCTAATATTTAGGCAAG	32.9
sh2 (= wt)	**------------------------------------**CAAGATGGAAAATA<br>AATTATATTAATTTTTGAAACCCATTAGGCATGCCTAATATTTAGGCAAG	37.4
sh3	**--**<br>----ATATTAATTTTTGAAACCCATTAGGCATGCCTAATATTTAGGCAAG	67.6
sh4	**--**<br>**-----------------------**ATTAGGCATGCCTAATATTTAGGCAAG	74.6
sh5	CTGCCATGCCTCAAGAACACCTTTGTTTTGCACTGACAAGATGGAAAATA<br>AATTATATTAATTTTTGAAACCCATTAGGC**--------------------**	71.6
1-7	**ACCTCGT**GAAAATAAATTATATTAATTTTTGAAACCCATTAGGCATGCCTAATATTTAGGCAAG	34.2
8-14	CAAGATG**TCCCCGC**AATTATATTAATTTTTGAAACCCATTAGGCATGCCTAATATTTAGGCAAG	55.3
15-21	CAAGATGGAAAATA**CCGGCGC**TTAATTTTTGAAACCCATTAGGCATGCCTAATATTTAGGCAAG	60.0
22-28	CAAGATGGAAAATAAATTATA**GGCCGGG**TTGAAACCCATTAGGCATGCCTAATATTTAGGCAAG	66.6
29-35	CAAGATGGAAAATAAATTATATTAATTT**GGTCCCA**CCATTAGGCATGCCTAATATTTAGGCAAG	29.9
36-42	CAAGATGGAAAATAAATTATATTAATTTTTGAAAC**AACGGCT**GCATGCCTAATATTTAGGCAAG	40.8
43-49	CAAGATGGAAAATAAATTATATTAATTTTTGAAACCCATTAG**TACGTAA**TAATATTTAGGCAAG	75.1
50-56	CAAGATGGAAAATAAATTATATTAATTTTTGAAACCCATTAGGCATGCC**GCCGCGG**TAGGCAAG	78.8
57-64	GAAGATGGAAAATAAATTATATTAATTTTTGAAACCCATTAGGCATGCCTAATATT**GCTTACCT**	80.0
MUTI	CAAGATGGAAAATAAATTATATTAATTTTTGAAACCCATTAGGCATGCCTAATA**GGG**AGGCAAG	60.1
MUTII	CAAGATGGAAAATA**CCGGCGC**TTAATTTTTGAAACCCATTAGGCATGCCTAATATTTAGGCAAG	73.3
MUTI/II	CAAGATGGAAAATA**CCGGCGC**TTAATTTTTGAAACCCATTAGGCATGCCTAATA**GGG**AGGCAAG	98.6
CDEImut	CAAGATGGAAAATAAATTATAT**A**AATTTTTGAAACCCATTAGGCATGCCTAATATTTAGGCAAG	66.9
CDEIImut	CAAGATGGAAAATAAATTATATTAATTTTTGAAACCCATTAGGCATGCCTA**C**TATTTAGGCAAG	72.8
CDEI/IImut	CAAGATGGAAAATAAATTATAT**A**AATTTTTGAAACCCATTAGGCATGCCTA**C**TATTTAGGCAAG	88.5
M1	CAAGATGGAAAATAAATT**T**TAT**A**AATTTTTGAAACCCATTAGGCATGCCTAATATTTAGGCAAG	37.4
M2	CAAGATGGAAAATAAATT**G**TAT**C**AATTTTTGAAACCCATTAGGCATGCCTAATATTTAGGCAAG	33.4
M3	CAAGATGGAAAATAAATT**C**TAT**G**AATTTTTGAAACCCATTAGGCATGCCTAATATTTAGGCAAG	38.6
M4	CAAGATGGAAAATAAATTATATTAATTTTTGAAACCCATTAGGCATGCCTA**T**TAT**A**TAGGCAAG	40.9
M5	CAAGATGGAAAATAAATTATATTAATTTTTGAAACCCATTAGGCATGCCTA**C**TAT**G**TAGGCAAG	35.2
M6	CAAGATGGAAAATAAATTATATTAATTTTTGAAACCCATTAGGCATGCCTA**G**TAT**C**TAGGCAAG	34.2
M7	CAAGATGGAAAATAAATT**G**TAT**C**AATTTTTGAAACCCATTAGGCATGCCTA**G**TAT**C**TAGGCAAG	36.6
M8	CAAGATGGAAAATAAATTATATTAATTTTTGAAACCCATTAGGCATGCCTA**G**TAT**U**TAGGCAAG	57.3
M9	CAAGATGGAAAATAAATTATATTAATTTTTGAAACCCATTAGGCATGCCTA**U**TAT**G**TAGGCAAG	55.8
M10	CAAGATGGAAAATAA**C**TTATATTAA**G**TTTTGAAACCCATTAGGCATGCCTAATATTTAGGCAAG	32.2
M11	CAAGATGGAA**G**ATA**C**ATT**G**TAT**C**AAT**G**TTTGAAACCCATTAGGCATGCCTAATATTTAGGCAAG	34.4
M12	CAAGATGGAAAATAAATTATATTAATTTTTGAAACCCATTAGGCATGC**G**TAATATTTA**C**GCAAG	33.1
M13	CAAGATGGAAAATAAATTATATTAATTTTTGAAACCCATTAGGCATGC**T**TAATATTTA**A**GCAAG	33.3
M14	CAAGATGGAAAATAAATTATATTAATTTTTGAAACCCATTAGGCATGCCT**T**ATATT**A**AGGCAAG	32.5

M15	CAAGATGGAAAATAAATTA**C**ATTAATTTTTGAAACCCATTAGGCATGCCTAATATTTAGGCAAG	41.2
M16	CAAGATGGAAAATAAATTATATTAATTTTTGAAACCCATTAGGCATGCCTAA**C**ATTTAGGCAAG	41.0
M17	CAAGATGGAAAATAAATTA**C**ATTAATTTTTGAAACCCATTAGGCATGCCTAA**C**ATTTAGGCAAG	46.3
M18	CAAGATGGAAAATAAATTAT**G**TTAATTTTTGAAACCCATTAGGCATGCCTAATATTTAGGCAAG	46.8
M19	CAAGATGGAAAATAAATTATATTAATTTTTGAAACCCATTAGGCATGCCTAAT**G**TTTAGGCAAG	51.4
M20	CAAGATGGAAAATAAATTAT**G**TTAATTTTTGAAACCCATTAGGCATGCCTAAT**G**TTTAGGCAAG	61.5
M21	CAAGATGGAAAATAAATTATA**C**TAATTTTTGAAACCCATTAGGCATGCCTAATATTTAGGCAAG	50.6
M22	CAAGATGGAAAATAAATTATATTAATTTTTGAAACCCATTAGGCATGCCTAATA**C**TTAGGCAAG	52.1
M23	CAAGATGGAAAATAAATTATA**C**TAATTTTTGAAACCCATTAGGCATGCCTAATA**C**TTAGGCAAG	73.7
M24	CAAGATGGAAAATAAATTATA**A**TAATTTTTGAAACCCATTAGGCATGCCTAATATTTAGGCAAG	54.0
M25	CAAGATGGAAAATAAATTATATTAATTTTTGAAACCCATTAGGCATGCCTAATA**A**TTAGGCAAG	45.6
M26	CAAGATGGAAAATAAATTATA**A**TAATTTTTGAAACCCATTAGGCATGCCTAATA**A**TTAGGCAAG	75.2
M27	CAAGATGGAAAATAAATTATA**G**TAATTTTTGAAACCCATTAGGCATGCCTAATATTTAGGCAAG	51.3
M28	CAAGATGGAAAATAAATTATATTAATTTTTGAAACCCATTAGGCATGCCTAATA**G**TTAGGCAAG	49.9
M29	CAAGATGGAAAATAAATTATA**G**TAATTTTTGAAACCCATTAGGCATGCCTAATA**G**TTAGGCAAG	80.3
M30	CAAGATGGAAAAT**C**AATTATATTAATTTTTGAAACCCATTAGGCATGCCTAATATTTAGGCAAG	50.2
M31	CAAGATGGAAAATAAATTATATTAATTTTTGAAACCCATTAGGCAT**C**CCTAATATTTAGGCAAG	73.1
M32	CAAGATGGAAAAT**C**AATTATATTAATTTTTGAAACCCATTAGGCAT**C**CCTAATATTTAGGCAAG	90.4
R1	**ATGAATGTACACTTATAGAAGAATTATGTAGGCTTACTGAACAATACATTAATCTGATGATAAC**	70.5
R2	**TAAAGAAGACTATAAAAGCGTGAAATATAAGTAGTTTACTCTACCTCAGGAATTATTTCTTGAA**	72.3
R3	**TTCGCGATAAAAGAACAGAGAACTAATAGGCATCATAAATTTGATCATGATATAATTTTAGTTC**	93.2
R4	**GACGTCTAGACAGCTAAAAAGCGATATTAAAAATGTAGCTTTAAGCTAATTTTATATCAATGAT**	91.3
R5	**GGTGATAATAAAGAATACTTTATCATAACACAGTTTTAGAAGTTTATCGCCGATTGACAAAAAT**	85.0
64 nt	**GCAAGTGTCCTTCGGTTATCTACGATCTTGACGCAGGCCACTTATGTGTGGGTGCCTACCCCCT**	97.2

* RLU are given in % relative to an empty vector control without *UCP3* 3’UTR sequences.

Supplementary Table 2. Oligonucleotide sequences used for reverse transcription and qPCR analysis.

**Specificity**	**Sequence 5' → 3'**
miR130_SL	GTTGGCTCTGGTGCAGGGTCCGAGGTATTCGCACCAGAGCCAAATGCCC
miR148_SL	GTTGGCTCTGGTGCAGGGTCCGAGGTATTCGCACCAGAGCCAACAAAG
miR152_SL	GTTGGCTCTGGTGCAGGGTCCGAGGTATTCGCACCAGAGCCACCAAGT
miR301_SL	GTTGGCTCTGGTGCAGGGTCCGAGGTATTCGCACCAGAGCCAGCTTTG
U48_SL	GTTGGCTCTGGTGCAGGGTCCGAGGTATTCGCACCAGAGCCAACGGTCAG
130a_fwd	CGGCGGCAGTGCAATGTTAAAA
130b_fwd	GGCGGCAGTGCAATGATGAAA
148a_fwd	GCGGTCAGTGCACTACAGAA
148b_fwd	AGCGGTCAGTGCATCACAGAA
152_fwd	GGCGGTCAGTGCATGACAGA
301a_fwd	GGCGGCAGTGCAATAGTATTGT
301b_fwd	GGCGGCAGTGCAATGATATTGT
U48_fwd	GAGTGATGATGACCCCAGGTAA
universal rev-primer	GTGCAGGGTCCGAGGT
GFP_qPCR_fwd1	CGTAAACGGCCACAAGTTCA
GFP_qPCR_rev1	CGCTACCCCGACCACATGAAG
RPLP0_fwd	TCGACAATGGCAGCATCTAC
RPLP0_rev	ATCCGTCTCCACAGACAAGG
DDIT4_F1	GTTTGACCGCTCCACGAG
DDIT4_R1	CATCAGGTTGGCACACAAGT
CYR61_F2	CGAGGTGGAGTTGACGAGA
CYR61_R2	GAGCACTGGGACCATGAAGT
PLAGL2_F1	GCAACCAGAGCAGAGACCAT
PLAGL2_R1	GTCCTTGCGGTGAAACATCT
SNAI1_F1	CTCTTTCCTCGTCAGGAAGC
SNAI2_R1	CGGTGGGGTTGAGGATCT
NRARP_F2	ATGACCAACTGCGAGTTCAA
NRARP_R2	GAACTTGACCAGCAGCTTCA
CSF3_F2	GTCCCACCTTGGACACACTG
CSF3_R2	GACACCTCCAGGAAGCTCTG
PLOD2_F1	GGGGCCAGAAAGTGAGATTA
PLOD2_R1	CCACTTTGTGGTTTGCCTTT

TNFSF15_F2	GCAGACGGAGATAAGCCAAG
TNFSF15_R2	GCCAGGCCTAGTTCATGTTC
ZC3H12A_F1	TCCACTCCCAGAAGAGGAAA
ZC3H12A_R1	GGATGGCACAAACACTGTGA
TM2D3_F1	GGGAAGCCTGTCACTTTTGA
TM2D3_R1	CCAGCAAAATCTGCAAGTCA
CASD1_F1	CATCACCTCCATAGCACCACT
CASD1_R1	TGCAGCTTCATTGTAAGCATCT
ITCH_fwd1	GAAGACGTTTGTGGGTGATT
ITCH_rev1	TAAGAAGCGGGGTTTATCTG
WWP1_fwd1	GACATGGAGATTTTGGGAAA
WWP1_rev1	CAAGGAAAGCTTTGGTCTGT
MAP3K5_fwd2	CAGGAAAACCCCCATTTTAT
MAP3K5_rev2	GCTCTCTTGTCAGGATCTGG
GTPBP8_fwd1	TGCAAATTCTTCACACATTTCT
GTPBP8_rev1	ATGGCTTTAGAGCACCTGAA
hsRC3H1_fwd	TGGACAACCAGAACCACAAA
hsRC3H1_rev	GCTGATCCATTTGGTACATCAC
hsRC3H2_fwd	AAGGTTGGCGCTAATGGTC
hsRC3H2_rev	CAGGAGTCTTGGGTGGAGAA
mmRC3H1_fwd	TTGTACCTGAAGCCACTCAGCAGT
mmRC3H1_rev	TCCACTAGCTGGCAATGAACCAGA
mmRC3H2_fwd	TGCCCATTCTCAGGAAGAGCTTGA
mmRC3H2rev	GCTGTGGTTGTGACAGTGCTGTTT
mmUCP3_fwd	GATGTGGTGAAGGTCCGATT
mmUCP3_rev	GGCATTTCTTGTGATGTTGGG

Supplementary Table 3. Conserved 3'UTR structures predicted by Dynalign and tested for functionality *in vivo* (related to **Supplementary Figure 1**).

Gene symbol	Chromosome location	Sequence 5' → 3'
ADM	chr11:10306992-10307091	GTGTCACCCCACCAGGGCGCAAGCCTCACTATTACTTGAACTTTCCAAAA<br>CCTAAAGAGGAAAAGTGCAATGCGTGTTGTACATACAGAGGTAACTATCA
BCL2L1	chr20:31665060-31665159	CCCCAGAGCTGTTTATGGCCTCAGCTGCCTCACTTCCTACAAGAGCAGCC<br>TGTGGCATCTTTGCCTTGGGCTGCTCCTCATGGTGGGTTCAGGGGACTCA
CTBP1	chr4:1211900-1211999	CATGAACGTTCTTGTCTGTGTACAGTTTTTAGAACATTACAAAGGATCTG<br>TTTGCTTAGCTGTCAACAAAAAGAAAACCTGAAGGAGCATTTGGAAGTCA
EGLN3	chr14:33925414-33925513	AAATGGAGGTGGTAGATGCCACAGAGAGGCATCACGGAAGCCTTAACAGC<br>AGGAAACAGAGAAATTTGTGTCATCTGAACAATTTCCAGATGTTCTTAAT
ELK3	chr12:96267231-96267330	CCCACGGGCTAGTTTACCTGTGTCGTGAGAAGGACATTGTGAAACTCTTG<br>TTAATTTGGTTTGCACTTTTCATAACATGGATAGTCTAGATTTATGTTAG
EMX1	chr2:72934005-72934104	GGCCCCTGCGTGGGCCCAAGCTGGACTCTGGCCACTCCCTGGCCAGGCTT<br>TGGGGAGGCCTGGAGTCATGGCCCCACAGGGCTTGAAGCCCGGGGCCGCC
HIRA	chr22:19330849-19330948	ATTGAATTTCCTTTGGCCGATAATCAGGATTTCCCTATAAGTCACTTGGA<br>CATTGGTCACTTGTAGGAAATTTAAACTCTAATTATGACAGCTACACTGA
HMOX1	chr22:35393699-35393798	GAAGGCTTTCAGGGCCTCCAGCCCTCTCACTGTGTCCCTCTCTCTGGAAA<br>GGAGGAAGGAGCCTATGGCATCTTCCCCAACGAAAAGCACATCCAGGCAA
HOXC9	chr12:54002875-54002974	TTTAGAGTTAGTTCTACCCAGCGAGGAGGAGGCGGGGAGAGAAACTGCGT<br>TCTCTTTCCCCAGCGCAACCGAAATAAATGACACATACAAATGTGATTTT
MYF6	chr12:80709011-80709110	CCTTCCTGGCCTAATCCTTTAGATTAGGTCACATTACATTAACATTTAGG<br>AACCCAGACCGAAAAGTTGCTGAAAGGGAAGGAGACACATTCACAAAGAA
NR4A2	chr2:156325469-156325568	TTTATACCACTGTATTGTGTGTAGTCCATGTTCTAAATCCAGGATGCCCC<br>GGAGCCAAAATGCCCTTTCAGGTTCTGCCTGCAGGTTAGGAAATAGCAAC
PDE5A	chr4:119498128-119498227	GAATGTTTGGAATCTTTCCTTTTTCAAAAGTAGGTTAGGAGCAAATTATC<br>ATACATTCTGTGACATTTAAAGCCTTTATAGGATAGTGAAAAATGCTGGC
RBPJ	chr4:26431008-26431107	CTACCGTCTTTTTGCTAGGACTTAAACTGACTTGAGTGTGGCAAAAAGTT<br>AACAAAAAAGGAGAAAAAATGAACAATCGTTTGTGGTTTCTTGGGAAAAC
SIX2	chr2:45005909-45006008	GTTCTTGTTTGGGATTTATTTTCAACAAGTTACTTTTAGGATCCTTTTGG<br>GGCTGGAGACTGAGTCTTGAACCACAGAAGGGAATAAATTATACACCACT
TFDP1	chr13:113641068-113641167	TGTTTACATACTTATAAGTCTATCATTTAAAGACATGTACTGAAACAAAT<br>GTATTTGTTTCATAAGCATCTTCCTGTAATCTATTATAAAATTGAAATTA
TGFBR1	chr9:99153356+99153455	TATTGTATTTGTGCAGGATTCTTTAGGCTTTATCAGTGTAATCTCTGCCT<br>TTTAAGATATGTACAGAAAATGTCCATATAAATTTCCATTGAAGTCGAAT
THBS1	chr15:39595970-39596069	ATGTAAATAGGCACTTAAATAGAAGCAGGAAAGGGAGACAAAGACTGGCT<br>TCTGGACTTCCTCCCTGATCCCCACCCTTACTCATCACCTGCAGTGGCCA
TLX3	chr5:171311950-171312049	TTTTTTCTTTAGAAACCGGCCACCTGCTTCCCCCGCGGGGGCCGCTGGAG<br>GAAGGGCAGCCGACCCGGCCGCTGGGGGAAGTGCCAGGGGCCCGGGGCAC
UBE2B	chr5:134390754-134390853	TGTTAAAATACATAACTTCAGTGCAAGAGACTTTGTCACTTATTTCCTTA<br>TGTGTGTAGGAGGGGTTAATAAGTCTCTAGCTCTCCATCTATTGATAGTT
UCP3	chr11:74001139-74001238	CTGCCATGCCTCAAGAACACCTTTGTTTTGCACTGACAAGATGGAAAATA<br>AATTATATTAATTTTTGAAACCCATTAGGCATGCCTAATATTTAGGCAAG

Supplementary Table 4. Cycle thresholds of miRNAs predicted to bind to the *UCP3* element.

	C_T value	
**hsa-miR-**	**HEK293**	**Hela**
148a	37.4	36.3
148b	36.8	35.9
152	36.9	35.8
130a	n.d.	n.d.
130b	36.6	36.4
301a	n.d.	n.d.
301b	n.d.	n.d.

n.d. = not detected

Supplementary Table 5. Proteins enriched by RNA affinity purification.

Description	Gene Symbol	MW [kDa]	No. of peptides				
			wt	CDEI mut	CDEII mut	CDEI/II mut	R2
Roquin-2	RC3H2	132	30	20	23	0	0
Zinc finger CCCH domain-containing protein 7A	ZC3H7A	111	19	0	29	0	0
Spermatid perinuclear RNA-binding protein	STRBP	72	14	10	11	0	0
Roquin-1	RC3H1	126	7	1	6	0	0
Gem-associated protein 5	GEMIN5	168	5	26	0	0	0
ATP-dependent RNA helicase DDX1	DDX1	74	5	12	15	0	0
RNA-binding protein MEX3A	MEX3A	54	3	0	5	0	0
tRNA-splicing ligase RtcB homolog	RTCB	55	4	4	8	0	0
RNA-binding protein 47	RBM47	64	2	0	8	0	0
RNA-binding protein Raly	RALY	32	3	5	0	0	0

Supplementary References

1. Regulski, E.E. and Breaker, R.R. (2008) In-line probing analysis of riboswitches. *Methods Mol Biol*, **419**, 53-67.
2. Mathews, D.H. (2005) Predicting a set of minimal free energy RNA secondary structures common to two sequences. *Bioinformatics*, **21**, 2246-2253.
3. Lu, J. (2008) Secondary structure prediction of non-coding RNA. Ph.D. Thesis, University of Rochester, School of Medicine & Dentistry, Rochester, N.Y., http://hdl.handle.net/1802/6609
4. Washietl, S., Hofacker, I.L. and Stadler, P.F. (2005) Fast and reliable prediction of noncoding RNAs. *Proc Natl Acad Sci U S A*, **102**, 2454-2459.
5. Mathews, D.H., Disney, M.D., Childs, J.L., Schroeder, S.J., Zuker, M. and Turner, D.H. (2004) Incorporating chemical modification constraints into a dynamic programming algorithm for prediction of RNA secondary structure. *Proc Natl Acad Sci U S A*, **101**, 7287-7292.
6. Macke, T.J., Ecker, D.J., Gutell, R.R., Gautheret, D., Case, D.A. and Sampath, R. (2001) RNAMotif, an RNA secondary structure definition and search algorithm. *Nucleic Acids Res*, **29**, 4724-4735.
7. Reuter, J.S. and Mathews, D.H. (2010) RNAstructure: software for RNA secondary structure prediction and analysis. *BMC Bioinformatics*, **11**, 129.
8. Sloma, M.F. and Mathews, D.H. (2017) Base pair probability estimates improve the prediction accuracy of RNA non-canonical base pairs. *PLoS Comput Biol*, **13**, e1005827.
9. Lu, Z.J. and Mathews, D.H. (2008) Fundamental differences in the equilibrium considerations for siRNA and antisense oligodeoxynucleotide design. *Nucleic Acids Res*, **36**, 3738-3745.
10. Karolchik, D., Hinrichs, A.S., Furey, T.S., Roskin, K.M., Sugnet, C.W., Haussler, D. and Kent, W.J. (2004) The UCSC Table Browser data retrieval tool. *Nucleic Acids Res*, **32**, D493-496.
11. Blanchette, M., Kent, W.J., Riemer, C., Elnitski, L., Smit, A.F., Roskin, K.M., Baertsch, R., Rosenbloom, K., Clawson, H., Green, E.D. *et al.* (2004) Aligning multiple genomic sequences with the threaded blockset aligner. *Genome Res*, **14**, 708-715.
12. Betel, D., Koppal, A., Agius, P., Sander, C. and Leslie, C. (2010) Comprehensive modeling of microRNA targets predicts functional non-conserved and non-canonical sites. *Genome Biol*, **11**, R90.
